# Effect of TAR hairpin stabilization on HIV-1 reverse transcription

**DOI:** 10.1128/jvi.01840-25

**Published:** 2026-04-29

**Authors:** Sandra Da Silva Amaral, Clémence Richetta, Lola Oualid, Nhat Quang Tu, Brigitte René, Olivier Mauffret, Olivier Delelis, Philippe Fossé

**Affiliations:** 1 LBPA, UMR8113 CNRS, ENS Paris-Saclay, Université Paris-Saclay 27048, Gif-sur-Yvette, France; University Hospital Tübingen, Tübingen, Germany

**Keywords:** HIV-1, reverse transcription, TAR, strand transfer, nucleocapsid protein, structure, DNA degradation

## Abstract

Reverse transcription of the human immunodeficiency virus type 1 (HIV-1) genome requires the first strand transfer that relies on a base-pairing interaction between the 3′ R sequence of the genomic RNA (gRNA) and the r sequence of the strong-stop DNA (ssDNA). The annealing reaction between the cTAR hairpin of ssDNA and the TAR hairpin of gRNA plays an essential role in r–3′ R pairing. Studies suggest that HIV-1 nucleocapsid protein (NC) has only a minor role in ssDNA synthesis but facilitates the annealing reaction by destabilizing the two complementary hairpins. We designed mutations to increase the stability of the cTAR DNA hairpin. These mutations should impair the NC-mediated annealing process, but not other viral processes. We investigated the effects of mutations in the annealing reaction, viral infectivity, and reverse transcription in infected cells. The mutations stabilizing the lower stem of the cTAR hairpin impair the annealing reaction, strongly reduce HIV-1 replication, and the amount of full-length ssDNA in infected cells. The *in vitro* and *ex vivo* data support NC as the essential player in the first strand transfer reaction by facilitating the initiation of the cTAR-TAR pairing through the zipper pathway. These results agree with previous studies suggesting a coevolutionary relationship between the stability of the cTAR hairpin and NC activity. Our data are consistent with the hypothesis proposed in an early work, suggesting that ssDNA degradation by cellular nucleases occurs when the first strand transfer cannot take place. This attractive hypothesis warrants further investigation in future studies.

**IMPORTANCE:**

The first strand transfer is a crucial step of the reverse transcription process. Because the great majority of first strand transfers in HIV-1 occur with full-length ssDNA, the annealing reaction between the cTAR DNA hairpin and the 3′ TAR RNA hairpin plays an essential role in the first strand transfer. To our knowledge, our study is the first to show that mutations stabilizing the lower stem of the cTAR hairpin strongly decrease the annealing reaction, HIV-1 replication, and the amount of full-length ssDNA in infected cells. Our data support the notion that NC is the essential player in the first strand transfer reaction by facilitating the initiation of the cTAR-TAR pairing through the zipper pathway.

## INTRODUCTION

HIV-1 replication depends on the progress of reverse transcription. Reverse transcription of the single-stranded viral genomic RNA (gRNA) corresponds to a sequence of steps leading to the synthesis of double-stranded DNA with an LTR at each end (1). An essential step of reverse transcription is the first strand transfer, which requires the presence of the R repeat sequence at both ends of the gRNA. This transfer primarily relies on a base-pairing interaction between the 3′ R sequence of gRNA and the r sequence of the strong-stop DNA (ssDNA). The ssDNA is synthesized by the polymerase activity of the reverse transcriptase (RT) when it copies the R-U5 region of the gRNA. During ssDNA synthesis, the degradation of the R-U5 region by the RNase H activity of RT is required for the first strand transfer because it releases the ssDNA (2). The model of reverse transcription suggests that the first strand transfer needs the full-length ssDNA (1). An early study on HIV-1 with mutations in the 5′ R sequence supports the notion that partial ssDNAs can be transferred to the 3′ R region before complete reverse transcription of the 5′ R element (3). However, these transfers occur at low frequencies (3). Furthermore, the results obtained by another site-directed mutagenesis study are consistent with the notion that the great majority of first strand transfers in HIV-1 occur with full-length ssDNA (4).

**Fig 1:**
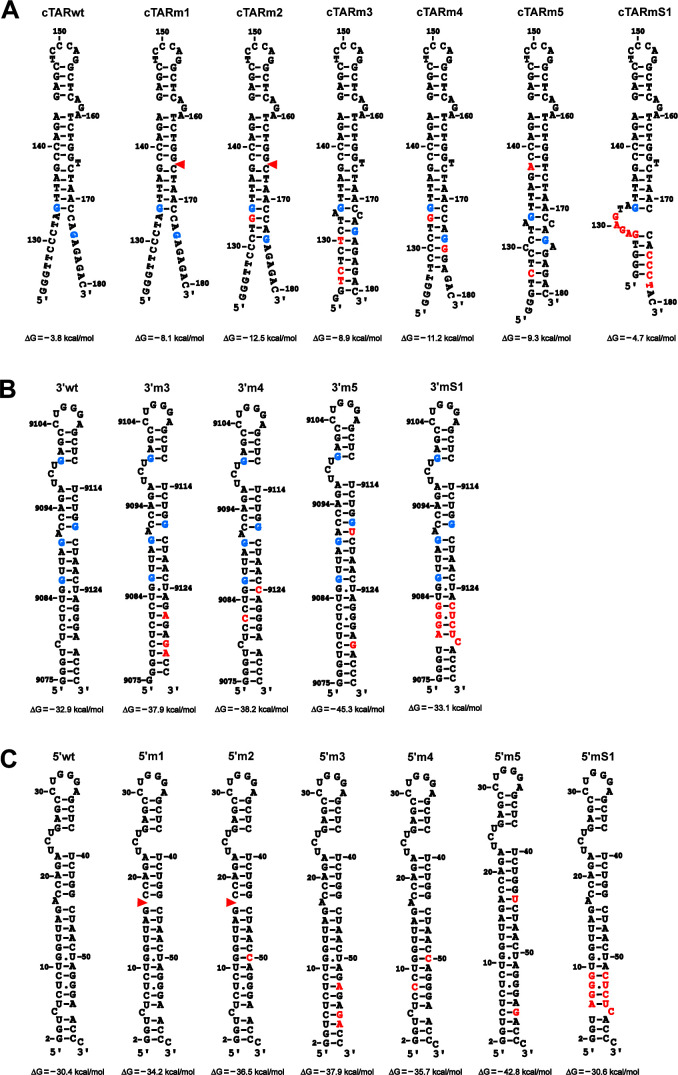
Predicted secondary structures for wild-type and mutated TAR sequences in the ssDNA (**A**), 3′ UTR (**B**), and 5′ UTR (**C**). The UNAFold web server (https://www.unafold.org/), based on the folding programs of Zuker (13), was used to calculate the free energies of structures. Mutations and deletions are shown as red letters and red triangles, respectively. The blue-colored guanine residues interact with NC (14, 15). (**A**) The free energies were calculated in the presence of 75 mM NaCl and 0.2 mM MgCl_2_. The numbering of the cTAR DNA hairpin corresponds to the ssDNA secondary structure (8). The sequence of cTAR does not possess two cytosine nucleobases at the 3′-end because it is likely that HIV-1 RT does not copy the 5′ cap into cytosine (16). (**B and C**) The folding and numbering of the 3′ and 5′ TAR RNA hairpins are according to the secondary structure of the HIV-1 genome (7) and alternative transcription start sites (17). G2 in the 5′ TAR hairpins corresponds to the 5′ cap (5′−5′-triphosphate-linked 7-methylguanosine) (17).

*In vitro* and *ex vivo* studies showed that the 5′ and 3′ R sequences of the HIV-1 gRNA form two hairpins named TAR and poly(A) because they contain the transactivator response element and the polyadenylation signal, respectively (5–7). In an *in vitro* study, we showed that the r sequence of the ssDNA forms the cTAR and cpoly(A) hairpins that are complementary to the TAR and poly(A) hairpins, respectively (8). Because the great majority of first strand transfers in HIV-1 occur with full-length ssDNA (4), the annealing reaction between the cTAR DNA hairpin and the 3′ TAR RNA hairpin plays an essential role in the first strand transfer. *In vitro* studies also suggest that the TAR/cTAR hairpins are more important for the first strand transfer than the poly(A)/cpoly(A) hairpins (9, 10). The wild-type cTAR hairpin in its ssDNA conformation contains single-stranded ends, two bulges, and no more than five consecutive Watson-Crick base pairs (8) (Fig. 1A). This “Y” conformation is consistent with the hypothesis that the 5′/3′ termini of complementary hairpins form a zipper intermediate that initiates the annealing reaction (11, 12).

The unfolding of the complementary hairpins is required to allow r–3′ R pairing corresponding to the DNA–RNA heteroduplex of 95 base pairs (18). To our knowledge, no studies have shown that RT, an essential player in reverse transcription, is capable of facilitating this pairing. *Ex vivo* and *in vitro* studies support the notion that HIV-1 nucleocapsid protein (NC), thanks to its nucleic acid chaperone activity, is involved in r–3′ R pairing (8, 18–22). This activity depends on three properties (23): (i) ability to aggregate nucleic acids; (ii) destabilization of short nucleic acid duplexes; and (iii) rapid on-off binding kinetics. NC binds preferentially to unpaired guanines and weakly base-paired G bases that are adjacent to wobble base pairs, mismatches, bulges, or loops (14, 15, 24–27). Bernacchi *et al*. (28) reported that NC destabilizes only one base pair of the TAR RNA hairpin, whereas it destabilizes seven to eight base pairs of the lower part of the cTAR DNA hairpin under similar experimental conditions. A single-molecule study using optical tweezers strongly suggests that NC destabilizes the TAR RNA hairpin by interacting with four paired guanines that are adjacent to low-stability regions (two bulges and one G-U wobble pair) (15). An NMR study showed that among these paired guanines, the G-C base pair located in the apical stem and adjacent to the three-nucleotide single-stranded bulge is destabilized by NC at a low concentration (27). In a previous *in vitro* study performed with ssDNA, we showed that the lower stem of the cTAR hairpin is more open (loss of four base pairs) in the presence of NC than in its absence (8). *In vitro* studies performed with short DNAs corresponding to the cTAR sequence have also shown that NC destabilizes the lower stem (28–30). The destabilizing effect of NC on the cTAR hairpin involves its binding to the G134 and G174 residues (14) (Fig. 1A). Destabilization of the cTAR hairpin by a truncated form of NC dramatically decreases when mutations allow the 5′- and 3′-ends to form a double-stranded region composed of 11 consecutive Watson-Crick base pairs (30). Recently, we showed that NC does not promote the formation of an RNA homoduplex from two RNA hairpins when the stem of the hairpins contains 10 consecutive Watson-Crick base pairs (31). These results support the notion that a stability threshold, determined by the nature and number of consecutive base pairs, restricts the destabilization activity of NC. A hypothesis is that there is an evolutionary relationship between the stability of the hairpins and the destabilizing activity of NC (30).

Two studies comparing the *in vitro* and *ex vivo* properties of NC mutants suggest that the nucleic acid chaperone activity of the NC is necessary during the reverse transcription of gRNA (20, 21). Because of the pleiotropic effects of NC mutations on the HIV-1 life cycle, the replication defects observed cannot be attributed solely to the decrease in the chaperone activity of NC (19, 20, 23). An earlier *in vitro* study (32) investigated the effect of removing bulges in the 5′ TAR hairpin (Fig. 1C) on ssDNA synthesis. This study showed that C5 bulge removal decreases the rate of ssDNA synthesis more strongly in the absence than in the presence of NC. In contrast, the 5′ TAR hairpin containing the C5 bulge and a stem of 24 consecutive base pairs only slightly reduces the synthesis of the full-length ssDNA (32). More recently, Brady *et al*. (33) showed that efficient synthesis of ssDNA in cells infected by HIV-1 requires DHX9/RNA helicase A (RHA). These authors proposed a model in which RHA unwinds the structured viral RNA and removes NC from the template, allowing for efficient production of ssDNA.

**Fig 2:**
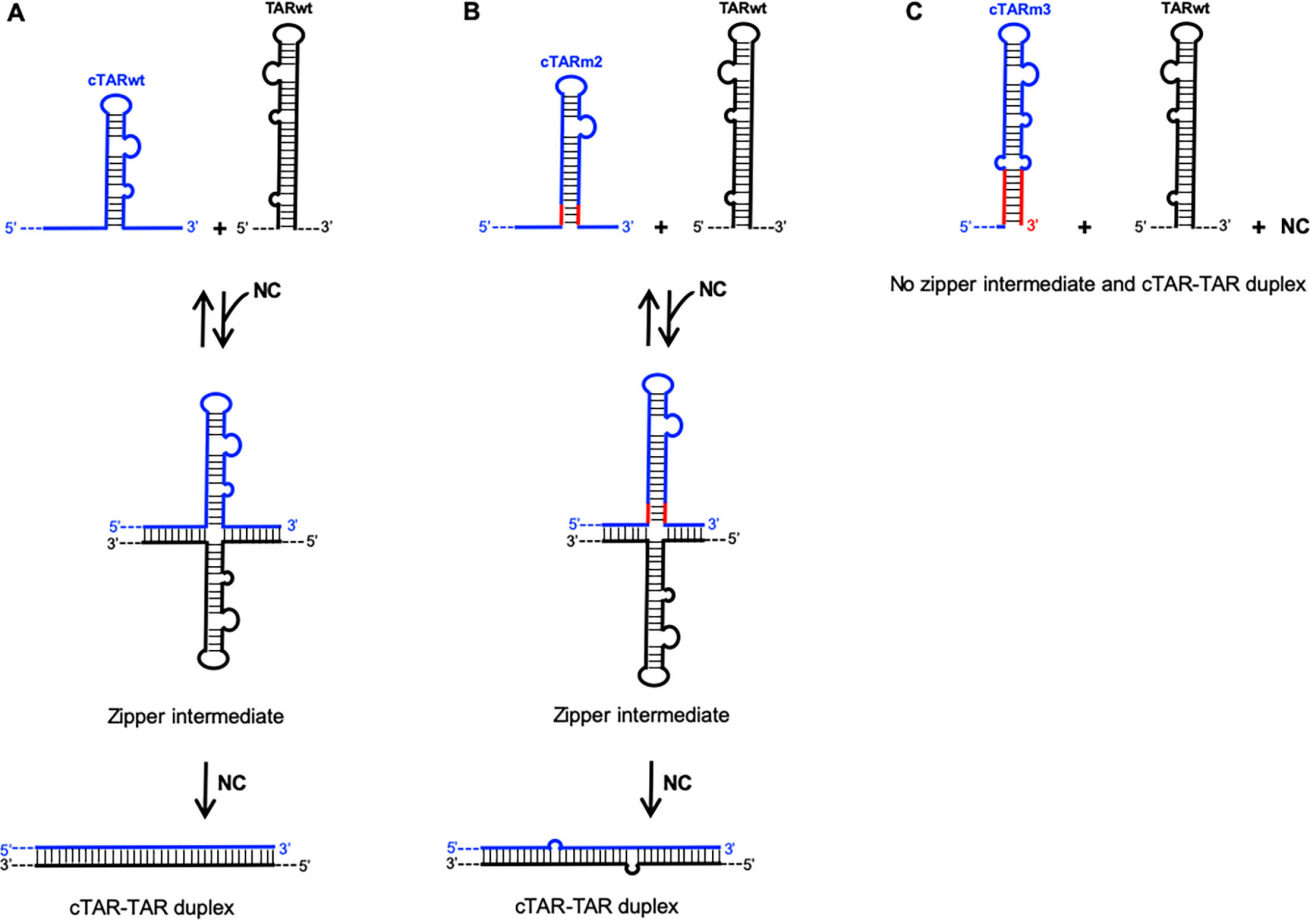
Effect on NC-mediated cTAR-TAR pairing of mutations stabilizing the cTAR hairpin. (**A**) The wild-type cTAR and TAR hairpins form the DNA–RNA heteroduplex through the zipper pathway. (**B**) Mutations do not prevent the formation of the heteroduplex when they stabilize the cTAR hairpin while leaving the 5′ and 3′ ends unpaired, allowing these ends to form the intermediate zipper (for example, the cTARm2–TARwt interaction). The red lines indicate the stem extension (three base pairs in the case of cTARm2). (**C**) Mutations (red lines) that stabilize and close the lower stem of the cTAR hairpin (e.g., the cTARm3) prevent the formation of the DNA–RNA heteroduplex through the zipper pathway. Diagrams are not to scale. The blue-dashed line represents the cU5 and cpoly(A) regions of ssDNA. The black-dashed lines at positions 5′ and 3′ represent the U3 and poly(A) regions of gRNA, respectively.

In summary, the various studies suggest that NC plays an essential role in the annealing reaction between the cTAR DNA hairpin and the TAR RNA hairpin but only a minor role in ssDNA synthesis. We first aimed to demonstrate a relationship between the first strand transfer *ex vivo* and the ability of NC to destabilize the cTAR stem-loop and pair it with the TAR stem-loop *in vitro*. In other words, the first objective was to obtain results that support NC as the essential player in cTAR-TAR pairing and, consequently, in the first strand transfer. To achieve this goal, we stabilized the cTAR stem-loop through various mutations, aiming to prevent its destabilization by NC and thereby inhibit the cTAR-TAR pairing required for the first strand transfer. We designed our site-directed mutagenesis strategy from the zipper model (Fig. 2A), which was proposed by *in vitro* studies (11, 12). We first stabilized the lower stem of the cTAR hairpin by mutations, while leaving its 5′ and 3′ ends unpaired (Fig. 2B). Second, we stabilized and closed the lower stem of the cTAR hairpin to prevent it from forming the zipper intermediate (Fig. 2C). The mutants were evaluated for the TAR DNA–RNA annealing reaction *in vitro*, viral infectivity, and reverse transcription in infected cells. The *in vitro* and *ex vivo* data support NC as the essential player in the first strand transfer reaction by facilitating the initiation of the cTAR-TAR pairing through the zipper pathway (Fig. 2A).

## RESULTS

### Design of cTAR mutants

We designed mutations that should increase the stability of the cTAR DNA hairpin and therefore impair the NC-mediated annealing process. We used the UNAFold web server (https://www.unafold.org/) based on the folding programs of Zuker (13) to calculate, in the presence of 75 mM NaCl and 0.2 mM MgCl_2_ (annealing conditions), the free energies of the structures presented in Fig. 1A. The cTARm2 hairpin is more stable than the other hairpins, although its ends are single-stranded. The high stability of the cTARm2 hairpin is mainly due to the deletion of the T bulge. Indeed, the insertion of the T bulge into the cTARm2 stem-loop leads to an increase in the free energy from −12.5 kcal/mol to −8.2 kcal/mol.

In the presence of NC, the average yield of the 3′ TAR RNA–cTAR DNA heteroduplex is not or only slightly reduced when the cTAR element contains not more than five mutations (34). Therefore, except for the cTARmS1 mutant, all our cTAR mutants contain one to three mutations (Fig. 1A). In other words, a negative effect of the studied mutations on the annealing reaction should not result from one to three mismatches, preventing the formation of a stable heteroduplex between the cTAR and 3′ TAR elements. The G134 and G174 residues likely play a role in the destabilizing effect of NC on the cTAR hairpin (14). G134 is adjacent to unpaired nucleotides, whereas G174 is unpaired. Interestingly, one (CCTAG) of the three putative high-affinity binding sites for NC in ssDNA contains G134 (8). Except for the cTARmS1 mutant, all mutants possess the two guanine residues. However, the local secondary structures containing the two residues in four mutants (m2, m3, m4, and m5) differ from that of the wild type (Fig. 1A). Deleting the T bulge generates a stem possessing 10 consecutive base pairs (cTARm1). This deletion and replacement of A133 by G133 reduce the length of the single-stranded ends and generate a stem containing 13 consecutive base pairs (cTARm2). Furthermore, G134 is not adjacent to unpaired nucleotides. Although the cTARm3 mutant possesses two bulges and one mismatch, its stability is higher than that of the wild-type because its ends form a double-stranded region composed of eight consecutive base pairs. In the cTARm3 hairpin, G134 is adjacent to an internal loop, whereas G174 is inside the lower stem. The cTARm4 and cTARm5 mutants contain 11 consecutive base pairs in the lower or central part. The G134 and G174 residues are inside the lower stem of the cTARm4 hairpin and adjacent to two internal loops in the cTARm5 hairpin. We used the mS1 mutant as a negative control because its mutations between nucleotides 174 and 177 strongly reduce reverse transcription in infected cells by leading to mismatches between the 3′-end of cTARmS1 DNA and the 5′-end of the wild-type 3′ TAR RNA (4). In addition, its nine mutations (five in the 5′ part and four in the 3′ part) are expected to impair the initiation of the annealing reaction through the zipper pathway (11, 12).

### Effect of stabilizing mutations on the TAR DNA–RNA annealing reaction

**Fig 3:**
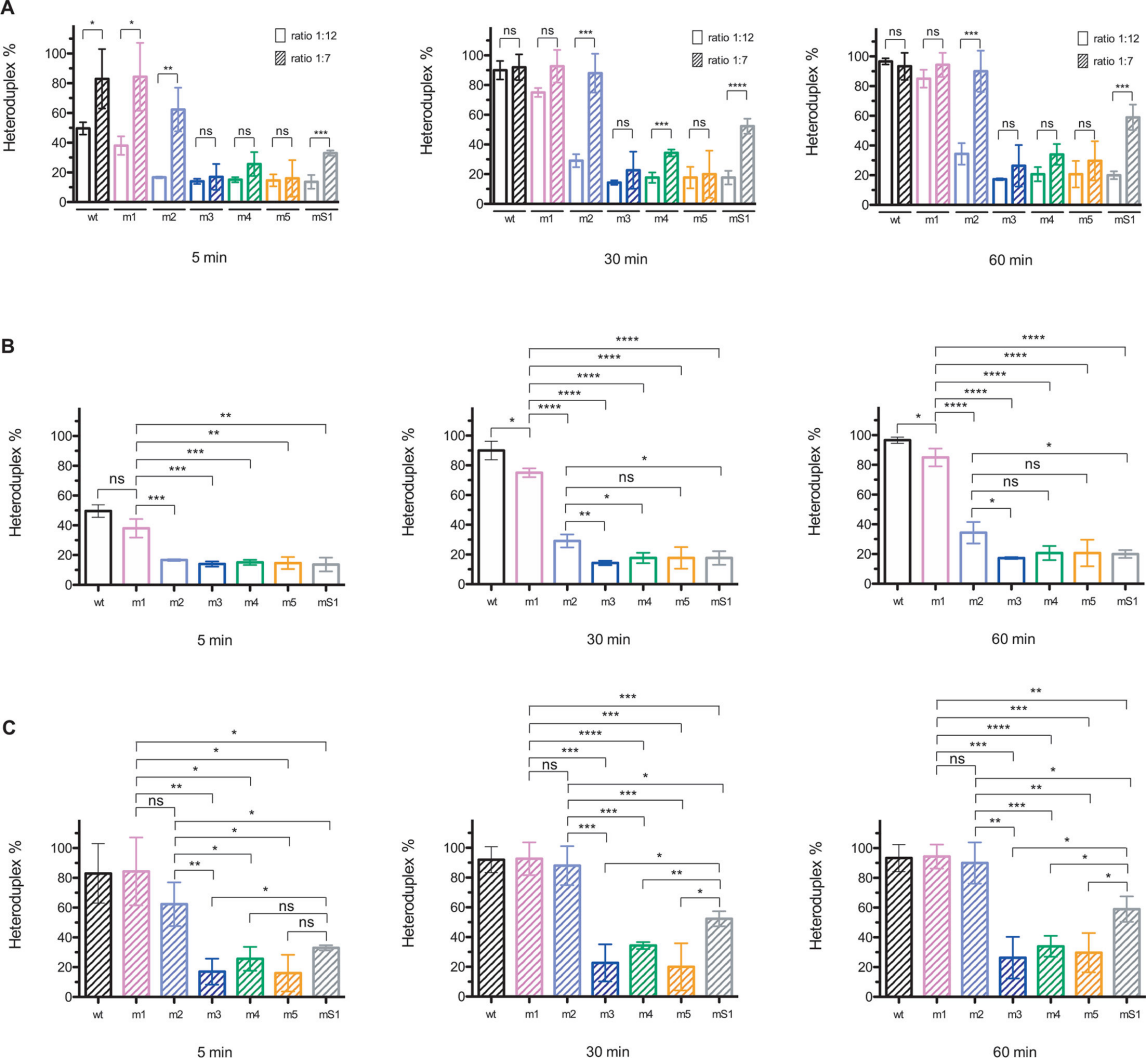
Effect of mutations on TAR DNA–RNA annealing in the presence of NC. The NC-mediated annealing assays were performed as described in Materials and Methods. (**A**) Comparison of percentages of cTAR–3′ TAR heteroduplexes generated by NC at protein to nucleotide molar ratios of 1:12 (empty bars) and 1:7 (hatched bars). (**B**) Percentages of cTAR–3′ TAR heteroduplexes generated by NC at a protein-to-nucleotide molar ratio of 1:12. At 5 min, there are no significant differences between the m2, m3, m4, m5, and mS1 mutants. At 30 and 60 min, there are no significant differences between the m3, m4, m5, and mS1 mutants. (**C**) Percentages of cTAR–3′ TAR heteroduplexes generated by NC at a protein-to-nucleotide molar ratio of 1:7. At 5, 30, and 60 min, there are no significant differences between the m3, m4, and m5 mutants. Data represent the mean ± SD of at least three independent experiments. *, *P* < 0.05; **, *P* < 0.01; ***, *P* < 0.005; ****, *P* < 0.001; ns, not statistically significant (unpaired two-samples *t*-test).

We investigated the annealing reaction using NC, the cTAR DNA mutants (Fig. 1A), and the 3′ TARwt RNA (Fig. 1B). The annealing assays were performed as described in Materials and Methods. These assays do not directly replicate viral conditions, as both protein and nucleic acid are significantly more dilute in these experiments than in the capsid (35). The experimental conditions used prevent the aggregation observed at high concentrations of nucleic acids and NC, while allowing for the effective pairing of the two complementary hairpins (11, 12). Because the TAR DNA/RNA elements are probably folded and associated with NC during the first strand transfer, they were renatured in the absence or presence of NC before being mixed. Annealing of 3′ TARwt RNA to the cTAR DNAs was barely detectable after 60 min incubation in the absence of NC (Fig. S1, lanes C2). The mature HIV-1 particles contain about 2000 NC molecules (35–37), that is, ~ one NC molecule for nine nucleotides. NC at a protein-to-nucleotide molar ratio of 1:7 efficiently promotes the annealing of strong-stop and cTAR DNAs to the 3′-end of the genomic RNA *in vitro* (8, 34). Therefore, we carried out the annealing assays at different times in the presence of NC at a protein-to-nucleotide molar ratio of 1:7 (Fig. S1C). In addition, we also performed the annealing assays at a protein-to-nucleotide molar ratio of 1:12 (Fig. S1A) to determine whether NC at a ratio lower than 1:9 is efficient in pairing the complementary hairpins. For the wt and m1 cTAR hairpins, a significant increase between the 1:12 and 1:7 ratios was observed at 5 min but not at other times (Fig. 3A). This observation shows that the maximum rate of heteroduplex was reached after 30 min incubation and NC at a protein-to-nucleotide molar ratio of 1:12 (Fig. 3A). Whatever the incubation time, the annealing rate for the m2 and mS1 cTAR hairpins was higher at the 1:7 ratio than at the 1:12 ratio (Fig. 3A), suggesting that destabilization of these hairpins is not efficient at the lowest ratio, which is lower than the ratio determined in the viral particle (see above). In contrast, the annealing rate for the m3, m4, and m5 cTAR hairpins did not significantly increase at the 1:7 ratio after 5 and 60 min of incubation, suggesting that destabilization of these hairpins is inefficient at the highest ratio.

The NC binding sites on the G134 and G174 residues of the cTARm1 hairpin should be in the same local secondary structure as in the wild-type hairpin (Fig. 1A). However, this mutant was slightly but significantly less efficient than the wild-type to form the TAR–cTAR heteroduplex after 30 and 60 min incubation at the 1:12 ratio (Fig. 3B; Fig. S1B), suggesting that the destabilizing activity of NC at this ratio is not optimal in the presence of a stem containing ten consecutive Watson-Crick base pairs. At the 1:12 ratio, the annealing rate decreased strongly with the other cTAR mutants (Fig. 3B; Fig. S1B). Interestingly, a single base substitution increased the stability of the DNA hairpin by extending its central stem by three base pairs (cTARm1 versus cTARm2). This stem extension strongly decreased the annealing reaction (Fig. 3B). These results suggest that NC at a protein-to-nucleotide molar ratio of 1:12 is inefficient in destabilizing a stem containing thirteen consecutive Watson-Crick base pairs. In addition, NC could hardly destabilize the cTARm2 hairpin because the G134 residue is not near unpaired nucleotides (Fig. 1A). There are no significant differences between the m3, m4, m5, and mS1 mutants at the 1:12 ratio (Fig. 3B). In contrast, the annealing rate was slightly higher with the m2 mutant than with the m3 and mS1 mutants, suggesting that NC cannot interact with the G174 residue in the m3 and mS1 hairpins because it is not adjacent to unpaired nucleotides in the m3 mutant and absent in the mS1 mutant.

**Fig 4:**
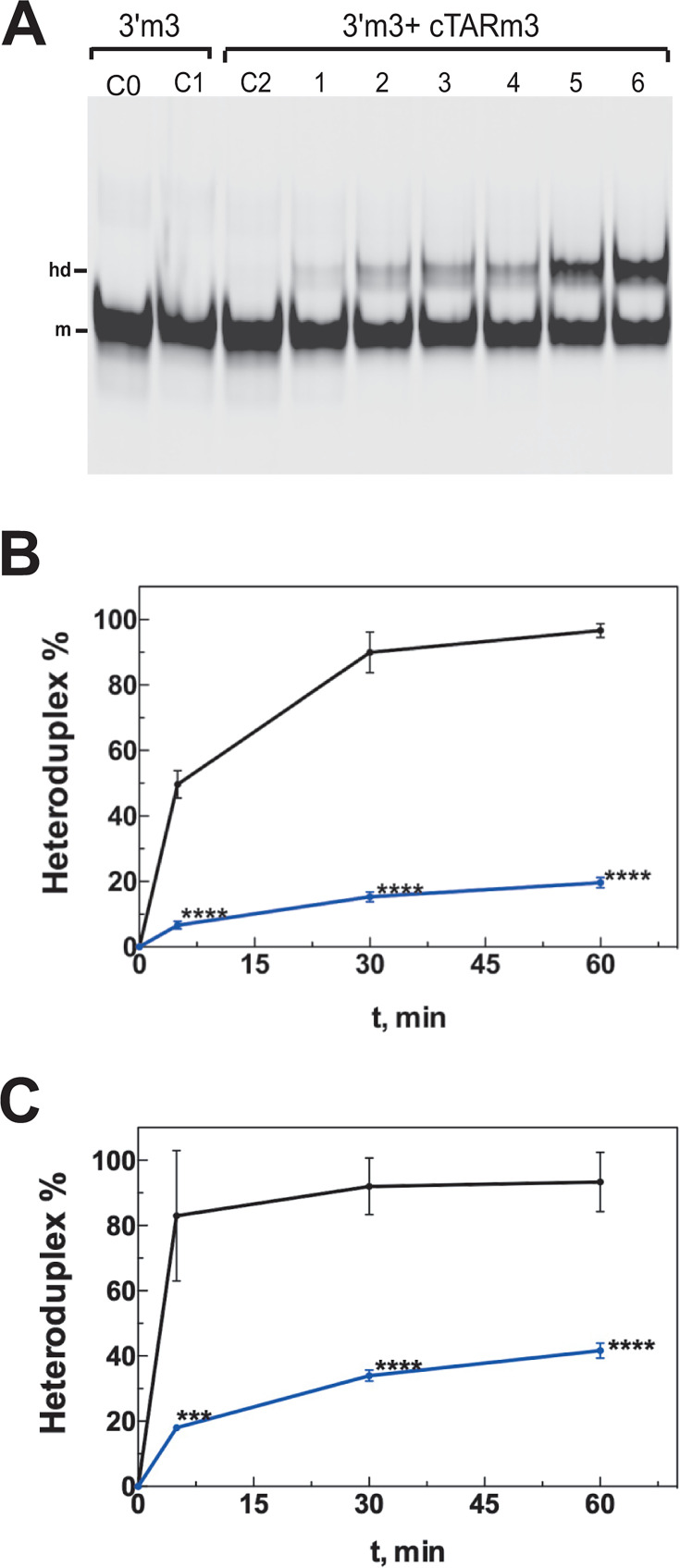
Time course of 3′ TARm3 RNA annealing with cTARm3 DNA in the presence of NC at various concentrations. The NC-mediated annealing assays were performed as described in Materials and Methods. (**A**) Assays with NC were carried out at a protein-to-nucleotide molar ratio of 1:12 (lanes 1–3) or 1:7 (lanes C1 and 4–6). Lane C0, a heat-denatured control, was used to identify the band corresponding to the monomeric 3′ TARm3 RNA that was labeled at its 5′-end by the Cy 5.5 fluorescent dye. Lane C2, the 5′-end labeled 3′ TARm3 RNA was incubated with cTARm3 DNA for 60 min in the absence of NC. The 5′-end labeled 3′ TARm3 RNA was incubated with cTARm3 DNA for 5 (lanes 1 and 4), 30 (lanes 2 and 5), or 60 min (lanes 3 and 6) in the presence of NC. The monomeric and heteroduplex forms of 3′ TARm3 are indicated by m and hd, respectively. (**B**) Percentage of the cTARm3–3′ TARm3 heteroduplex in the presence of NC at a protein-to-nucleotide molar ratio of 1:12. Black line, TARwt with cTARwt; blue line, TARm3 with cTARm3. Data represent the mean ± SD of at least three independent experiments. ***, *P* < 0.005; ****, *P* < 0.001 (unpaired two-samples *t*-test). (**C**) Percentage of the cTARm3–3′ TARm3 heteroduplex in the presence of NC at a protein-to-nucleotide molar ratio of 1:7.

At the 1:7 ratio, the m1 and m2 mutations did not significantly impair the annealing reaction (Fig. 3C; Fig. S1D). These results confirm that the deletion of T166 and a single base substitution (G133) does not reduce the ability of the cTARm2 hairpin to form a heteroduplex with the 3′ TARwt RNA. In contrast, the four other cTAR mutants significantly decreased the annealing rate. At the 1:7 ratio and after 30 and 60 min of incubation (Fig. 3C), the annealing rate of cTARmS1, forming a heteroduplex containing nine mismatches with the 3′ TARwt, was significantly higher than that of the cTARm3, m4, and m5 mutants harboring two or three mutations, leading to the formation of heteroduplexes containing two to three mismatches. These results are consistent with the notion that the NC-mediated annealing process depends in part on the stability of the complementary hairpins. Indeed, the cTARm3, m4, and m5 hairpins are predicted to be more stable than the cTARmS1 hairpin (Fig. 1A). To confirm this notion, the annealing time courses were performed with the cTARm3 and 3′ TARm3 mutants in the presence of NC at protein to nucleotide molar ratios of 1:12 and 1:7 (Fig. 4A). These mutants are predicted to form complementary hairpins that display high stability (Fig. 1A and B). At the 1:12 ratio and after 60 min of incubation, the average yields of the cTARm3–3′ TARm3 and cTARwt–3′ TARwt heteroduplexes were 19.7% and 96.7%, respectively (Fig. 4B). Even at the 1:7 ratio and after 60 min of incubation, the average yield of the cTARm3–3′ TARm3 heteroduplex (41.7%) was significantly lower than that of the wild-type heteroduplex (93.3%) (Fig. 4C). Taken together, our results are consistent with the notion that the designed mutations exert their deleterious effect on the annealing reaction by increasing the stability of the cTAR stem-loop, and not by destabilizing the cTAR–TAR heteroduplex through mismatches.

### Effect of stabilizing mutations on HIV-1 infection

To generate viruses expressing GFP and the cTAR mutations described above, we introduced the mutations into the 5′ TAR sequence (Fig. 1C) of an HIV-1 reporter virus described in Materials and Methods. Note that the 5′ LTR is responsible for GFP expression. Among the six mutant hairpins, two (m3 and m5) could decrease the full-length ssDNA synthesis because they do not possess the C5 bulge in the lower stem (32). However, efficient ssDNA synthesis in infected cells requires the unwinding activity of RHA that does not rely on the presence of bulges (33). Indeed, RHA can unwind an RNA–RNA duplex composed of 28 consecutive base pairs (33, 38). Therefore, it seems unlikely that stabilization of the 5′ TAR RNA hairpin by our mutations would decrease viral DNA synthesis. The mutations should impair only the first strand transfer step of the reverse transcription process. The mutations did not target the nucleotides involved in the twinned transcriptional start usage (17, 39). We did not mutate the upper part of the 5′ TAR hairpin located between nucleotides 18 and 44 because it is critical for transcription (40, 41). Although an early *in vitro* study suggests that the 5′ TAR element could constitute an RNA dimerization site (42), a more recent *in vitro* study shows that it does not (43). In addition, mapping of the HIV-1 RNA genome in infected cells and virions did not identify the 5′ TAR sequence as a dimerization site (44). However, the opening of the 5′ TAR hairpin causes aberrant RNA dimerization and packaging (45). In contrast, our mutations stabilize the 5′ TAR hairpin (Fig. 1C) and should not alter the dimerization and packaging processes. The wild-type 5′ TAR RNA hairpin possesses three bulges and eleven consecutive base pairs, of which two are wobble base pairs (Fig. 1C). The deletion of the A bulge generates a stem possessing 16 consecutive base pairs (mutants m1 and m2) whose stability increases by replacing the G-U wobble pair with the G-C base pair (mutant m2). The lower stem of the 5′ TARm3 mutant does not possess the C bulge and is composed of 15 consecutive base pairs. The stability of the 5′ TARm4 RNA hairpin is higher than that of the wild-type 5′ TAR RNA hairpin because the C-G/G-C base pairs replace the two wobble base pairs. In the 5′ TARm5 RNA hairpin, two base pairs replace two bulges and generate a stem of 21 consecutive base pairs. The stability and secondary structure of the 5′ TARmS1 RNA hairpin are similar to that of the wild type.

The HIV-1 reporter viruses were co-transfected into HEK293T cells with a vector bearing the VSV-G envelope gene to produce HIV-1 env−gfp+ viruses pseudotyped with the VSV-G envelope. All the mutant viral particles contained levels of CAp24 that were similar to those of the wild-type (data not shown). To confirm that the 5′ TAR mutations do not impair the packaging process, we quantified the genomic RNA (gRNA) from equivalent amounts of CAp24 by RT-qPCR. As expected, the amount of packaged gRNA in the mutant viral particles was not significantly different from that of the wild-type particles (Fig. S2A). Note that the efficient gRNA packaging by the 5′ TARmS1 mutant has also been reported (46).

**Fig 5:**
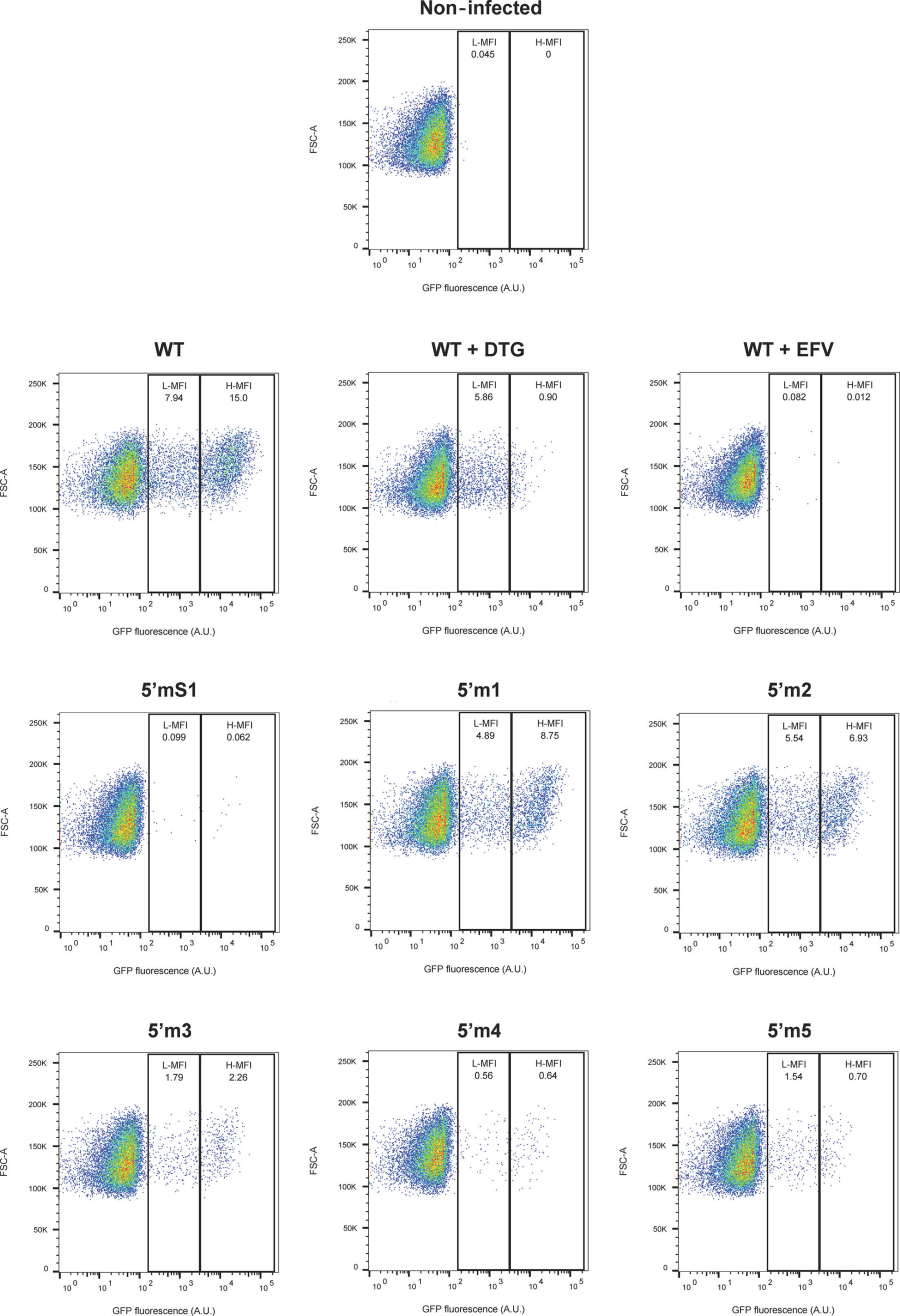
Gating strategy for flow cytometry analysis of GFP expression after infection with the wild-type virus or the 5′ TAR mutant viruses. MT4 T cells were infected with either HIV-1 *env^-^ gfp^+^* harboring the wild-type TAR sequences or a mutant 5′ TAR sequence, each pseudotyped with VSV-G protein, in the absence of antiretroviral drugs or presence of dolutegravir (wt+DTG) or efavirenz (wt+EFV). Two days later, flow cytometry was used to determine the percentage of GFP+ cells and the mean fluorescence intensity (MFI) of GFP expression. L-MFI: low MFI, H-MFI: high MFI. Dot plots show GFP fluorescence (expressed in arbitrary units, A.U.) depending on the Forward-Scatter parameter (FSC). Data are representatives of independent experiments.

We infected MT4 T-cells with HIV-1 env−gfp+ viruses pseudotyped with the VSV-G envelope and containing a wild-type or a mutant 5′ TAR sequence. We performed a flow cytometric analysis of GFP transgene expression in the infected cells. This highly sensitive method allows the direct analysis of viral expression in target cells after a single round of infection (47–49). The percentage of cells expressing the GFP transgene and their mean fluorescence intensity (MFI) was determined 1, 2, and 3 days postinfection. An illustrative experiment on day 2 is shown in Fig. 5. The fluorescence intensity of cells displayed a bimodal distribution. High mean fluorescence intensity (H-MFI) results from the strong expression of integrated viral DNA (47–49). In contrast, low mean fluorescence intensity (L-MFI) results from the weak expression of unintegrated viral DNA since dolutegravir (DTG), an integrase inhibitor, efficiently inhibits the presence of H-MFI cells (Fig. 5, 15% H-MFI cells versus 0.90% H-MFI cells). The inhibitory effect of DTG was also observed with the six 5′ TAR mutants on days 2 and 3 (Fig. S3A). In the absence of DTG, except for the 5′ TARm5 mutant on day 2, the relative percentages of H-MFI cells infected by the mutants were close to those of H-MFI cells infected by the wild type (Fig. S3B). Whatever the virus, the relative percentage of H-MFI cells strongly decreased in the presence of DTG (Fig. S3B). Therefore, these results show that the 5′ TAR mutations did not impair the integration process and its sensitivity to an integrase inhibitor.

**Fig 6:**
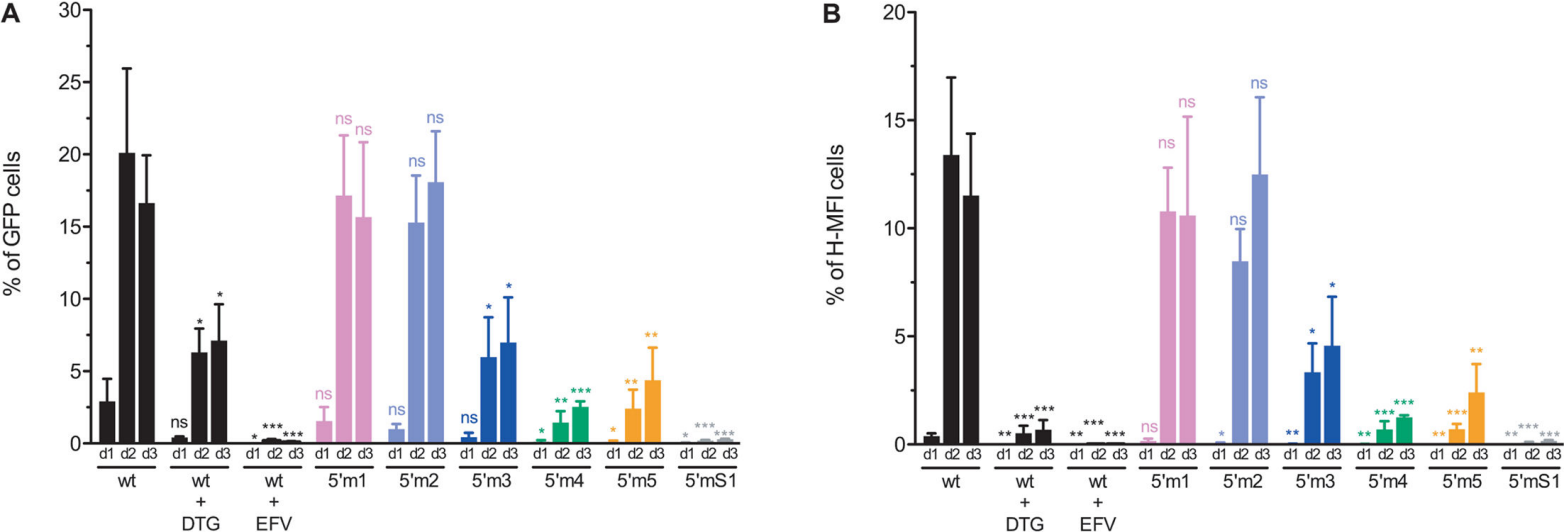
Time course of GFP expression. MT4 T-cells were infected with either HIV-1 *env^-^ gfp^+^* harboring the wild-type TAR sequences or a mutant 5′ TAR sequence, each pseudotyped with VSV-G protein, in the absence of antiretroviral drugs or in the presence of dolutegravir (wt+DTG) or efavirenz (wt+EFV). One (d1), 2 (d2), and 3 (d3) days postinfection, flow cytometry was used to determine the percentage of GFP+ cells and mean fluorescence intensity (MFI) of GFP expression. (**A**) Percentage of GFP+ cells (percentage of L-MFI cells + percentage of H-MFI cells). (**B**) Percentage of cells displaying H-MFI. Data represent the mean ± SD of at least three independent experiments. Means were compared with those of the wild type. *, *P* < 0.05; **, *P* < 0.01; ***, *P* < 0.005; ns, not statistically significant (unpaired two-samples *t*-test).

In the presence of the efavirenz (EFV) control (Fig. 5, panel wt + EFV), a reverse transcriptase inhibitor, the L-MFI and H-MFI cells were barely detectable (less than 0.1% positive cells), indicating that GFP expression requires the synthesis of viral DNA by the reverse transcription process. We obtained a similar result when the cells were infected with the 5′ TARmS1 mutant used as a control (Fig. 5, panel 5′mS1) because it is defective for reverse transcription and infectivity (4). The results presented in Fig. 5 also show that the populations of GFP-positive cells (L-MFI and H-MFI cells) were higher when the infection occurred with the m1 or m2 mutant than with the m3, m4, or m5 mutant. We analyzed the results of at least three independent experiments, 1, 2, and 3 days postinfection, to accurately evaluate the effect of 5′ TAR mutations on GFP expression (Fig. 6). The results obtained with the cells infected by the 5′ TARmS1 mutant or the wild-type virus in the presence of an RT inhibitor confirmed the assertions mentioned above. Altogether, our results obtained *in vitro* (Fig. S1D) are in good accordance with those obtained *ex vivo*. These *ex vivo* results show that the 5′ TARm1 and m2 mutants are not defective for reverse transcription. In contrast, the m3, m4, and m5 mutations significantly decreased the populations of GFP-positive cells (Fig. 6). Furthermore, the distribution between L-MFI and H-MFI is similar among all mutants, indicating a defect in the overall reverse transcription step for the m3, m4, and m5 mutants, rather than in the integration step. These results are consistent with the notion that mutations stabilizing the cTAR hairpin impair the first strand transfer reaction and subsequently the reverse transcription process.

**Fig 7:**
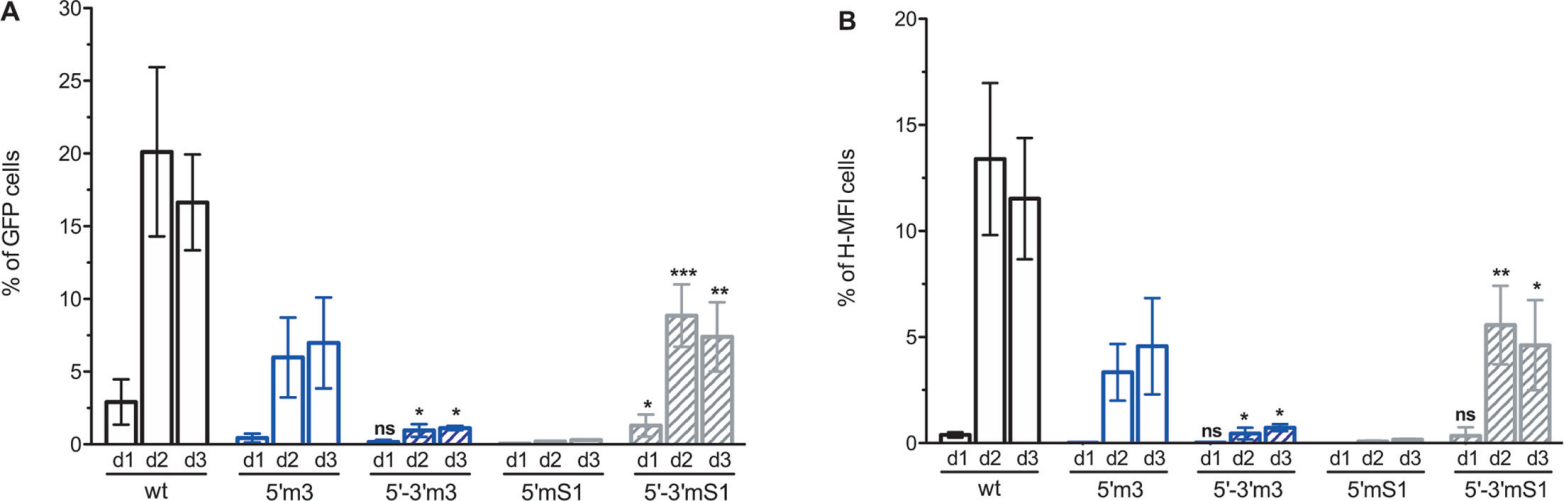
Comparison of infectivities of single and double TAR mutants. MT4 T-cells were infected with either HIV-1 *env^-^ gfp^+^* harboring the wild-type TAR sequences or a single/double mutant TAR sequence, each pseudotyped with VSV-G protein. One (d1), 2 (d2), and 3 (d3) days postinfection, flow cytometry was used to determine the percentage of GFP+ cells and mean fluorescence intensity (MFI) of GFP expression. (**A**) Percentage of GFP+ cells (percentage of L-MFI cells + percentage of H-MFI cells). (**B**) Percentage of cells displaying H-MFI. Data represent the mean ± SD of at least three independent experiments. Means of single mutants were compared with those of double mutants (5′m3 versus 5′−3′m3 and 5′mS1 versus 5′−3′mS1). *, *P* < 0.05; **, *P* < 0.01; ***, *P* < 0.005; ns, not statistically significant (unpaired two-samples *t*-test).

Likely, the deleterious effect of 5′ TARm3, 5′ TARm4, and 5′ TARm5 mutations did not result from mismatches between nucleotides 124 and 138 of the mutant cTAR sequence and nucleotides 9,119 and 9,133 of the wild-type 3′ TAR sequence (Fig. 1). Indeed, mutations in the 43–55 part of the 5′ TAR sequence generating mutations in the 128–140 part of the cTAR sequence introduce mismatches that do not affect the efficiency of reverse transcription (4). Reverse transcription is sensitive to mutations between nucleotides 2 and 10 of the 5′ TAR sequence, generating relatively short mismatches of three base pairs between the 172 and 180 part of the mutant cTAR sequence and the 9,077 and 9,085 part of the wild-type 3′ TAR sequence (4). In the 2–10 region, the 5′ TARm3 and 5′ TARm5 mutants do not contain mutations, whereas the 5′ TARm4 mutant contains a single-point mutation. To exclude the possibility that mismatches are responsible for the deleterious effect of 5′ TARm3, 5′ TARm4, and 5′ TARm5 mutations, we constructed four double mutants (5′−3′m3, 5′−3′m4, 5′−3′m5, and 5′−3′mS1), containing the mutations in both the 5′ and 3′ TAR elements. These double mutants cannot form mismatches between the cTAR sequence of the ssDNA and the TAR sequence at the 3′-end of the genomic RNA. The lower stem of the 3′ TAR hairpin was preserved in the double mutants (Fig. 1B) because its opening indirectly affects the polyadenylation of the viral transcripts (50). To check that the double mutations do not impair the packaging process, we quantified the gRNA from equivalent amounts of CAp24 by RT-qPCR (Fig. S2B). As expected, the amount of packaged gRNA in the 5′−3′m3 and 5′−3′mS1 particles did not decrease. The m3 double mutant was even twice more efficient than the wild-type for gRNA packaging. Note that the efficient gRNA packaging by the mS1 double mutant has also been reported (4). In contrast, the m4 and m5 double mutants packaged 5-fold less gRNA than the wild-type. These two last double mutants were not analyzed by flow cytometry, as we expected a significant decrease in the percentage of GFP-positive cells, resulting mainly from the packaging defect and not directly from an impairment in reverse transcription. Ohi and Clever (4) showed that the deleterious effect of 5′ TARmS1 mutations on infectivity/reverse transcription resulting from mismatches between the 3′-end of cTARmS1 DNA and the 5′-end of the wild-type 3′ TAR RNA does not occur when the mS1 mutations are present at both ends of gRNA. This observation is consistent with the notion that the double mS1 mutant can form a perfect heteroduplex between the cTARmS1 DNA and the 3′ TARmS1 RNA. As expected, the populations of GFP-positive cells were significantly higher when the infection occurred with the 5′−3′mS1 double mutant than with the 5′mS1 single mutant (Fig. 7). Conversely, the populations of GFP-positive cells were significantly lower when the infection occurred with the 5′−3′m3 double mutant than with the 5′m3 single mutant (Fig. 7). In all cases, the distribution between L-MFI and H-MFI was comparable, indicating no influence on the integration step. These results are consistent with the notion that the deleterious effect of 5′m3 mutations on GFP expression is not due to the presence of mismatches but to the stability of the cTARm3 hairpin. Likely, the stability of the 3′ TARm3 hairpin of the double mutant is responsible for the increased deleterious effect of m3 mutations on GFP expression.

### Effect of stabilizing mutations on HIV-1 reverse transcription

**Fig 8:**
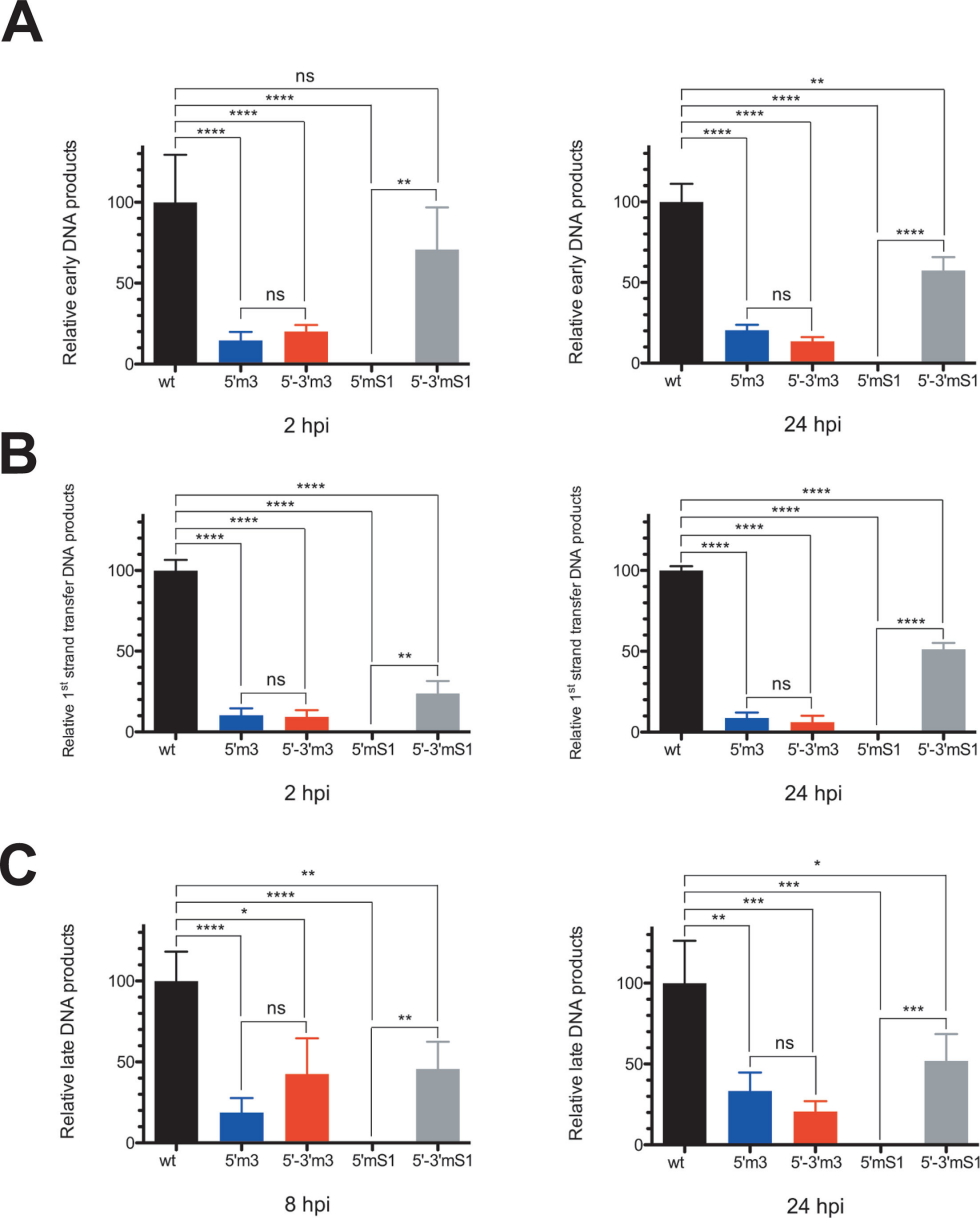
Effect of single and double TAR mutations on reverse transcription. MT4 T cells were infected with equivalent amounts of wild-type or mutant viruses as described in Materials and Methods. At 2, 8, or 24 h postinfection (2, 8, or 24 hpi), cells were harvested for DNA extraction, and HIV-1 cDNAs were measured by qPCR. (**A**) Graphs show the levels of early (strong-stop) DNA. (**B**) Graphs show the levels of minus-strand DNA transfer (first strand transfer). (**C**) Graphs show the levels of late DNA (second strand transfer). The levels of DNA products were normalized to the wild-type (arbitrarily set to 100%). Data represent the mean ± SD of at least three independent experiments. *, *P* < 0.05; **, *P* < 0.01; ***, *P* < 0.005; ****, *P* < 0.001; ns, not statistically significant (unpaired two-samples *t*-test).

To determine which step in reverse transcription was affected by the m3 and mS1 mutations, we investigated the ability of four mutants (5′m3, 5′−3′m3, 5′mS1, and 5′−3′mS1) to reverse transcribe. The infected MT4 T cells were harvested, and the DNA was extracted and analyzed by qPCR for early (strong-stop), minus-strand transfer (first strand transfer), and late (second strand transfer) viral DNA products (Fig. 8). We used a protocol that does not discriminate between non-integrated and integrated viral DNA. However, this does not pose a problem for our study, as the effect of mutations on DNA amount is already observable over short periods (2 and 4 h after infection, Fig. 8; Fig. S4), that is, under conditions where viral DNA corresponds only to the non-integrated form (51, 52).

The results obtained with the mS1 mutants are consistent with those of Ohi and Clever, yielded by a semi-quantitative PCR analysis (4). Indeed, we found significant amounts of ssDNA in the cells infected with the 5′−3′mS1 double mutant but not with the 5′mS1 single mutant (Fig. 8A), although the stability of the TARmS1 hairpin is similar to that of the wild-type (Fig. 1C). It is therefore difficult to propose a mechanism that would prevent the synthesis of the full-length ssDNA of the 5′mS1 single mutant. The most likely hypothesis proposed first by Ohi and Clever (4), based in part on the disrupted-virion endogenous reverse transcription assay, is that the ssDNA is degraded by cellular nucleases when its 3′ end cannot anneal with the 3′ end of the viral genome.

The cells infected by the 5′m3 single mutant contained approximately 7-fold and 5-fold less ssDNA, at 2 and 24 hpi, respectively, than those infected by the wild-type virus. The cells infected with the 5′m3 single mutant and the 5′−3′m3 double mutant contained similar amounts of ssDNA. Therefore, the perfect complementarity between the cTARm3 and 3′ TARm3 sequences does not allow for increasing the amount of ssDNA in the infected cells. These results, combined with those of annealing experiments, support a first hypothesis that the lower stem of the cTARm3 hairpin, composed of eight base pairs (Fig. 1A), reduces the annealing reaction and triggers ssDNA degradation. In other words, ssDNA degradation would occur when the 3′-end of this nucleic acid cannot pair with the 5′-end of the 3′ TAR hairpin. A second hypothesis is that the ssDNAm3 is not identified by qPCR because RT does not synthesize the cTAR portion. We do not favor the latter hypothesis because RHA promotes RT activity in the elongation step of ssDNA synthesis and can unwind an RNA–RNA duplex composed of 28 consecutive base pairs (33). We also analyzed the rate of ssDNA in cells infected by the 5′m4 and 5′m5 mutants (Fig. S4) because the cTARm4 and cTARm5 hairpins are not efficient for the annealing reaction (Fig. 3) and contain unpaired nucleotides at their extremities (Fig. 1). The cells infected by the 5′m4 and 5′m5 mutants contained approximately 3-fold less ssDNA at 2 hpi than those infected by the wild-type virus. The difference was between 20-fold and 25-fold less ssDNA at 4 and 8 hpi (Fig. S4). It seems unlikely that the low ssDNA level observed with the 5′m4 mutant resulted from a decrease in ssDNA synthesis by RT. Indeed, in the absence of NC and RHA, ssDNA synthesis is not decreased when the 5′ TAR stem-loop retains the C5 bulge and has a stem consisting of seventeen consecutive base pairs (32). Taken together, the results are consistent with the first hypothesis, that is, ssDNA degradation.

In agreement with the amounts of ssDNA, we found significant amounts of minus-strand DNA transfer products in the cells infected with the 5′−3′mS1 double mutant but not with the 5′mS1 single mutant (Fig. 8B). Consistent with the decrease in ssDNA, the cells infected by the 5′m3 single mutant contained approximately 10-fold and 11-fold less minus-strand DNA transfer products, at 2 and 24 hpi, respectively, than those infected by the wild-type virus. The cells infected with the 5′m3 single mutant and the 5′−3′m3 double mutant contained similar amounts of these reverse transcription intermediates generated by the first strand transfer (Fig. 8B). As expected and shown in Fig. 8C, the amounts of late DNA products in the cells infected by the wild-type virus and the mutant viruses were compatible with those observed for the reverse transcription intermediates (ssDNA and first-strand transfer products).

## DISCUSSION

The first strand transfer, requiring a base-pairing interaction between the 3′ TAR RNA hairpin of gRNA and the cTAR hairpin of the ssDNA, is a crucial step in the reverse transcription of gRNA (4, 9, 10). NC promotes cTAR–3′ TAR pairing *in vitro* (12, 27, 29). Unfolding of the two complementary hairpins is necessary to allow this pairing interaction. Many *in vitro* studies suggest that NC is essential for TAR DNA–RNA annealing by destabilizing the cTAR hairpin (8, 14, 15, 27–30, 53). A truncated form of NC is inefficient in destabilizing the mutant cTAR hairpin possessing 11 consecutive Watson-Crick base pairs in its lower stem (30). To our knowledge and until the present study, the cTAR–3′ TAR pairing has not been directly investigated in the presence of mutations stabilizing the cTAR hairpin. At different times and in the presence of NC at protein to nucleotide molar ratios of 1:12 and 1:7, we analyzed the annealing reaction between the 3′ TARwt RNA hairpin and six cTAR DNA mutant hairpins predicted to be more stable than the wild-type cTAR hairpin (Fig. 1A). The 1:12 ratio is lower than the 1:9 ratio estimated in the mature HIV-1 particle (35–37). The 1:7 ratio allows efficient annealing of cTAR DNA or ssDNA to the 3′-end of gRNA in *vitro* and could correspond to the local concentration of NC during the first strand transfer (8, 34). We found that the maximum rate of heteroduplex was reached with the complementary wild-type hairpins after 30 min of incubation and NC at a protein-to-nucleotide molar ratio of 1:12 (Fig. 3A). Our results obtained after 60 min of incubation with the cTARmS1 mutant used as a control show that the average yield of the 3′ TARwt–cTARmS1 heteroduplex containing nine mismatches is low (20%) at the 1:12 ratio and moderate (59%) at the 1:7 ratio (Fig. 3A). This heteroduplex likely involves a base-pairing interaction between nucleotides 132 and 173 of cTARmS1 and 9,084 and 9,125 of 3′ TARwt (Fig. 1). Formation of heteroduplex cannot be initiated through the zipper pathway because the 5′- and 3′-ends of cTARmS1 contain several successive mutations. Because the TAR DNA–RNA annealing reaction with the wild-type complementary hairpins can initiate through two pathways (zipper and loop-loop kissing) (12), the loop-loop kissing would be the pathway used by the cTARmS1 mutant. NC at a protein-to-nucleotide molar ratio of 1:7 efficiently promotes the formation of stable 3′ TAR RNA–cTAR DNA heteroduplexes containing one to five mismatches (34). Consistent with these results, we found that the average yield of the 3′ TARwt–cTARm2 heteroduplex containing two mismatches is similar to that of the wild-type heteroduplex in the presence of NC at a protein-to-nucleotide molar ratio of 1:7 (Fig. S1D). Thus, the annealing reaction at the 1:7 ratio does not decrease with the cTARm2 sequence, which can form the more stable DNA hairpin (Fig. 1A) and the zipper intermediate (Fig. 2B). In contrast, the average yield of heteroduplexes containing two or three mismatches and formed with the cTARm3, cTARm4, or cTARm5 mutants strongly decreased (Fig. S1D). Note that these mutants can form hairpins having a lower stability than the cTARm2 hairpin. Therefore, there is no direct relationship between the effect of mutations and the global cTAR hairpin stability. Interestingly, the cTARm3, cTARm4, or cTARm5 hairpins are less efficient than the cTARmS1 hairpin in forming a heteroduplex with the 3′ TARwt hairpin at the 1:7 ratio after 30 min of incubation (Fig. 3C). Therefore, the loop-loop kissing pathway is likely more impaired with these three stem-loops than the cTARmS1 stem-loop, which contains not more than five consecutive base pairs. The presence of one segment containing between 8 and 11 successive base pairs in the three mutant hairpins could contribute to the decrease in the annealing reaction via the loop-loop kissing and zipper pathways. Moreover, the destabilizing effect of NC on these hairpins could be reduced because their local secondary structure at the level of G134 and G174 residues differs from that of the wild-type hairpin (Fig. 1A). The extremities of cTARm3, cTARm4, or cTARm5 hairpins probably do not favor the annealing reaction via the zipper pathway because they contain few unpaired nucleotides and at least one mutation. In addition, the cTARm3 hairpin was inefficient in forming a heteroduplex with the complementary TARm3 hairpin (Fig. 4). This result is consistent with the notion that NC cannot efficiently destabilize the 15 consecutive base pairs in the lower stem of TARm3 (Fig. 1B). Moreover, this TAR mutant does not possess the C5 bulge that plays an essential role in the TAR lower stem metastability and terminal fraying (54). The cTARm1 and cTARm2 hairpins display a secondary structure that is compatible with the initiation of the annealing reaction through the two pathways. At the 1:12 ratio, the annealing rate decreased slightly with the cTARm1 mutant and highly with the cTARm2 mutant (Fig. S1B), suggesting that the destabilizing activity of NC at this ratio varies from high to low in the presence of long stems containing 10 (in the case of cTARm1) to 13 (in the case of cTARm2) consecutive Watson-Crick base pairs. The annealing rates of the cTARwt, cTARm1, and cTARm2 hairpins were similar at the 1:7 ratio (Fig. S1D). These results suggest that NC at a protein-to-nucleotide molar ratio of 1:7 can efficiently destabilize long DNA stems containing up to 13 consecutive Watson-Crick base pairs. Finally, the results show that the m3, m4, and m5 mutations are the most efficient in decreasing the cTAR-TAR pairing because by stabilizing the lower stem of the cTAR hairpin, they prevent the initiation of the annealing reaction through the zipper pathway (Fig. 2C).

To investigate the effect of mutations stabilizing the cTAR hairpin in infected cells, we introduced the mutations into the 5′ TAR sequence (Fig. 1C) of an HIV-1 reporter virus expressing GFP (47). As mentioned in the previous sections, the six mutant 5′ TAR RNA hairpins should not decrease the full-length ssDNA synthesis because RHA facilitates reverse transcription by unwinding RNA–RNA duplexes containing up to 28 base pairs (33, 38). We designed the mutations to impair only the first strand transfer step of the reverse transcription process, especially the cTAR–3′ TAR interaction. Consistent with their design, all the 5′ TAR mutations (Fig. 1C) do not impair the transcription and packaging processes because the amount of packaged gRNA in the mutant viral particles was not significantly different from that of the wild-type particles (Fig. S2A). Furthermore, using the d2 data from Fig. 7 and the qPCR data from Fig. 8C (right panel, 24 hpi), we checked that GFP expression is proportional to the amount of viral DNA measured by qPCR (Fig. S5A). We also confirmed that the number of H-MFI cells, where integrated viral DNA is expressed, is also proportional to the amount of viral DNA (Fig. S5B). These results rule out the possibility that our mutations decrease GFP expression by reducing TAR-Tat interaction-dependent transcription.

The reporter virus allows the direct analysis of HIV-1 expression in MT4 T cells during a single replication round. The appearance of fluorescence in the target cells shows the translation of the GFP transgene that results from several steps of the virus life cycle (reverse transcription, transcription, and splicing). Furthermore, high mean fluorescence intensity (H-MFI) reflects the strong GFP expression from integrated viral DNA (47–49). As expected, GFP expression dramatically decreased when the EFV reverse transcriptase inhibitor or the 5′ TARmS1 mutations strongly reduced the reverse transcription process (Fig. 6). Using the DTG integrase inhibitor, we showed that the 5′ TAR mutations do not impair the integration process and its sensitivity to an integrase inhibitor (Fig. S3B). Our results clearly show that the 5′ TARm1 and 5′ TARm2 mutants are not defective for GFP expression and, therefore, for reverse transcription. Thus, the m1 and m2 mutations in the 5′ TAR and cTAR hairpins cannot prevent the synthesis of the full-length ssDNA and the first strand transfer. These results support the notion that efficient synthesis of ssDNA in cells infected by HIV-1 involves RHA that can unwind stable RNA secondary structures. Moreover, they are consistent with the *in vitro* assays showing that NC at a protein-to-nucleotide molar ratio of 1:7 efficiently promotes the annealing of 3′ TARwt RNA hairpin to the cTARm1/m2 DNA hairpin (Fig. S1D). Consistent with the deleterious effect of the m3, m4, and m5 mutations on the cTAR–3′ TAR pairing (Fig. 3) and the amount of ssDNA (Fig. S4), GFP expression significantly decreased when we infected the MT4 T cells with the HIV-1 reporter virus bearing the 5′ TARm3, 5′ TARm4, or 5′ TARm5 hairpin (Fig. 6).

Except for the 5′m4 and 5′mS1 (negative control for reverse transcription) mutants, the *ex vivo* effect of our mutants is not due to the formation of mismatches, as these are not in the 2–10 region of the 5′ TAR element. Indeed, Ohi and Clever (4), using several mutants, showed that mismatches outside this region do not affect reverse transcription. More precisely, they showed that viruses are defective for reverse transcription when there are mismatches between the 3′-end of cTAR (nucleotides 174–179) and the 5′-end of 3′ TAR (nucleotides 9,078–9,083). In contrast, mismatches between the 5′-end of cTAR (nucleotides 128–135) and the 3′-end of 3′ TAR (nucleotides 9,122–9,129) do not impair the reverse transcription process. To confirm that mismatches between the mutant cTAR hairpin and the wild-type 3′ TAR hairpin are not responsible for the deleterious effect of mutations on GFP expression, we constructed four double mutants (5′−3′m3, 5′−3′m4, 5′−3′m5, and 5′−3′mS1), containing the mutations in both the 5′ and 3′ TAR elements. The m4 and m5 double mutants were not analyzed for GFP expression, as they packaged five times less gRNA than the wild-type (Fig. S2B). Because the mutations should not change the folding of the RNA packaging domain, we cannot propose a rational explanation for this surprising result. In agreement with an earlier study (4), GFP expression was much higher when the infection occurred with the 5′−3′mS1 double mutant than with the 5′mS1 single mutant (Fig. 7). In the same study, semi-quantitative PCR analysis showed that the amounts of reverse transcription intermediates (early, first strand transfer, and late DNAs) in the cells infected with the 5′−3′mS1 double mutant and the 5′mS1 single mutant were high and barely detectable, respectively. We found similar results using qPCR to analyze viral DNAs in infected cells (Fig. 8). Interestingly, the 5′mS1 virions are not defective for DNA synthesis in an endogenous reverse transcription assay (4). From these results completed by other 5′ and 3′ TAR mutants, Clever and Ohi (4) proposed that mismatches between the 3′-end of cTAR and the 5′-end of 3′ TAR induce rapid degradation of ssDNA by cellular nucleases. Our results support this hypothesis. Because the viral particles can incorporate host proteins such as uracil DNA glycosylase-2 and apurinic/apyrimidinic endonuclease (55, 56), we cannot rule out the possibility that they contain DNases capable of degrading the strong-stop DNA, whose action is restricted by the numerous NC molecules that protect nucleic acids by binding to them (57). Mutations preventing the cTAR-TAR pairing could increase the strong-stop DNA lifetime and thus its accessibility to DNases.

Three-prime repair exonuclease 1 (TREX1), which plays a crucial role in suppressing the interferon response during HIV-1 infection (58), is a 3′−5′ exonuclease that may trigger ssDNA degradation. Indeed, this enzyme efficiently processes DNA 3′-termini with three mismatched 3′-overhang (59). Interestingly, a small but detectable amount of TREX1 is mobilized to the nucleus upon HIV-1 infection, whereas this exonuclease is absent in the nuclei of uninfected cells (60). Several recent studies (61) strongly suggest that the completion of reverse transcription and HIV-1 capsid uncoating occurs in the nucleus rather than in the cytoplasm. An attractive hypothesis is that the 3′-end of the cTARmS1 hairpin of ssDNA is accessible to TREX1 because it contains three unpaired nucleotides (Fig. 1A), cannot pair with the 5′-end of 3′ TAR (four mismatches), and is therefore not extended by HIV-1 RT. The 3′-end of the cTARm4 hairpin also contains three unpaired nucleotides that TREX1 could recognize. However, ssDNA amount was low but higher with the m4 mutant than with the mS1 mutant (Fig. S4). An explanation is that NC would destabilize the lower stem of the cTARm4 hairpin of some ssDNA molecules. More precisely, these ssDNA molecules would not possess three unpaired nucleotides at their 3′-end and would be extended by the RT because a single mismatch would not abolish the annealing of the 3′-end of the cTARm4 hairpin with the 5′-end of the 3′ TARwt hairpin.

GFP expression was lower in the cells infected with the 5′−3′m3 double mutant than with the 5′m3 single mutant (Fig. 7). This result is compatible with the observation that the amounts of reverse transcription intermediates (early, first strand transfer, and late DNAs) were low in the cells infected with the 5′−3′m3 double mutant or the 5′m3 single mutant (Fig. 8). As mentioned above, the cTARm3 hairpin is inefficient in forming a heteroduplex with either the TARwt or the TARm3 hairpin (Fig. 3 and 4). In addition, the cTARm3 hairpin does not possess unpaired nucleotides at its 3′-end (Fig. 1A), and the cTARm3–3′ TARwt heteroduplex cannot form mismatches between the 3′-end of DNA and the 5′-end of RNA. Therefore, rapid degradation by TREX1 of ssDNAm3 seems unlikely. The annealing, GFP expression, and qPCR (early DNA) results obtained with the single mutant m5 are close to those obtained with the single mutant m3 (Fig. 6; Fig. S1 and S4) and therefore lead to the same hypothesis concerning TREX1.

Since the annealing reaction is inefficient with the m3 and m5 mutants (Fig. S1), the lifetime of the cTAR hairpin of these mutants is likely higher than that of the cTARwt hairpin in the infected cells. A hypothesis is that a DNA repair system recognizes DNA hairpins with long lifetimes as aberrant replication intermediates. This recognition would lead to the degradation of DNA hairpins by nucleases. The amounts of reverse transcription intermediates (early, first strand transfer, and late DNAs) were either low in the cells infected with the 5′m3 single mutant or not detectable with the 5′mS1 single mutant (Fig. 8). These observations are not surprising because annealing of the cTARm3 hairpin to the TARwt hairpin is low but not null (Fig. 3), and the 3′-end of cTARm3 does not form mismatches with the 5′-end of 3′ TARwt (Fig. 1). In other words, RT can extend the 3′-end of ssDNAm3. In contrast, RT cannot extend the 3′-end of ssDNAmS1 because four consecutive mismatches prevent the formation of a heteroduplex between the 5′-end of 3′ TARwt and the 3′-end of cTARmS1. The amount of ssDNA in cells infected by the 5′m5 mutant (Fig. S4) suggests that the behavior of this mutant is similar to that of the 5′m3 mutant but not to the 5′mS1 mutant.

Finally, our study is the first to investigate the effect of stabilization of the cTAR hairpin *in vitro* and in infected cells. We show that mutations stabilizing the lower stem of the cTAR hairpin impair the annealing reaction required for the first strand transfer and strongly reduce the amount of full-length ssDNA in infected cells. Our results are consistent with previous studies suggesting a coevolutionary relationship between the cTAR structure and NC activity, promoting the first strand transfer (8, 14, 30). More precisely, the TAR DNA–RNA annealing process, necessary to the first strand transfer, requires that the 5′ and 3′ extremities of the cTAR hairpin do not form a stable stem to be able to initiate the annealing reaction via the zipper pathway (Fig. 2A). Cellular nucleases would degrade ssDNA possessing a stable hairpin at its 3′-end. In another study, it would be interesting to determine whether TREX1 is responsible for the degradation of some mutated ssDNA.

## MATERIALS AND METHODS

### Nucleocapsid protein

HIV-1 nucleocapsid protein (NC(1–55)) was synthesized by the Fmoc/opfp chemical method and purified to homogeneity by HPLC, as described previously (8).

### Oligonucleotides

DNA/RNA oligonucleotides used in gel-shift annealing assays and site-directed mutagenesis by PCR were purchased from Eurogentec. DNA oligonucleotides and probes used in qPCR were purchased from TIB Molbiol.

### Plasmid constructs

Subcloning procedures introduced all mutations into the previously described NLENG1-ES-IRES vector (16,150 bp), which originates from the HIV-1 NL4-3 genome (see the diagram presented in the additional file 1 of reference 47). The pLF1 plasmid (5,267 bp) containing the 5′ wild-type R sequence was generated using the pCG44 plasmid (62), and the PCR insert was produced from the NLENG1-ES-IRES vector and the OXho and OEco primers (Table S1). The 5′ R mutations were generated using pLF1-specific primers (Table S1) and PCR-based site-directed mutagenesis. The PCR products were digested with SacI and intramolecularly ligated to produce six pLF1 mutants (pLF1-m1, pLF1-m2, pLF1-m3, pLF1-m4, pLF1-m5, and pLF1-mS1). The six pLevD5 mutants (pLevD5-m1, pLevD5-m2, pLevD5-m3, pLevD5-m4, pLevD5-m5, and pLevD5-mS1) containing the 5′ R mutations were constructed by subcloning the AatII-BssHII fragment of the pLF1 mutants into the NLENG1-ES-IRES vector cleaved by the same endonuclease restriction enzymes. The pEP2 plasmid (5,940 bp) containing the 3′ wild-type R sequence was generated using PCR, the OXFW and OXREV primers (Table S1), and the NLENG1-ES-IRES vector. The 3′ R mutations were generated using pEP2-specific primers (Table S1) and PCR-based site-directed mutagenesis. The PCR products were digested with SacI and intramolecularly ligated to produce four pEP2 mutants (pEP2-m3, pEP2-m4, pEP2-m5, and pEP2-mS1). The four pLevD3 mutants (pLevD3-m3, pLevD3-m4, pLevD3-m5, and pLevD3-mS1) containing the 3′ mutations were constructed by subcloning the XhoI-NgoMIV fragment of the pEP2 mutants into the NLENG1-ES-IRES vector cleaved by the same endonuclease restriction enzymes. Each pLevD5-3 mutant harboring mutations in both the 5′ and the 3′ R sequences was generated by swapping the XhoI-NgoMIV fragment of pLevD3 containing the 3′ R mutation into the pLevD5 containing the 5′ R mutation. All mutations were confirmed by Sanger sequencing (Eurofins Genomics).

### Gel-shift annealing assay

Two 3′ TAR RNAs (3′wt and 3′m3 in Fig. 1B) were labeled at their 5′-terminus by Cy 5.5, which is a fluorescent dye (absorbance 675 nm; emission 694 nm), whereas the cTAR DNAs (Fig. 1A) were not labeled. The annealing assay was carried out in a final volume of 12 µL. The concentrations used for nucleic acids (250 nM) and NC (1.2 µM or 2.0 µM) were of the same order of magnitude as those used in two studies focusing on the TAR-cTAR interaction (11, 12). The 5′-end labeled TAR RNA (1 pmol) in 4 µL of water was heated at 90°C for 2 min and chilled for 2 min on ice. Then, 1 µL of renaturation buffer (final concentrations: 75 mM KCl, 0.2 mM MgCl_2_, 1 mM DTT, and 50 mM Tris-HCl, pH 7.8) and 1 µL of NC (4.9 or 8.4 pmol) were added and the sample was incubated at 37°C for 15 min. Unlabeled cTAR DNA (2 pmol) underwent the same renaturation treatment with 1 µL of NC (9.5 or 16.3 pmol) and then added to the refolded TAR RNA. The protein to nucleotide molar ratios were 1:12 or 1:7. These ratios refer to total nucleotide concentration. The reaction mixture was then incubated at 37°C for 5, 30, or 60 min. At the end of incubation, 1 µL of stop solution (9% SDS, 50 mM EDTA) was added and the assay was phenol/chloroform extracted. The aqueous phase was mixed with 4 µL of loading buffer (50% wt/vol glycerol, 0.05% wt/vol bromophenol blue, and 0.05% wt/vol xylene cyanol). The samples were analyzed by electrophoresis on a 12% polyacrylamide gel (37.5:1 [wt/vol], acrylamide/bisacrylamide) at 20°C in the TBE buffer (89 mM Tris-borate [pH 8.3], 2 mM EDTA). After the electrophoresis, the monomeric (m) and heteroduplex (hd) forms of TAR RNA were visualized and quantified using the Amersham Typhoon^TM^ 5 device and Image-Quant software (Cytiva, France). The percent of heteroduplex was determined as 100 × (hd/(hd + m)).

### Cells

Human HEK293T (ATCC CRL-11268) and MT-4 cells (63) were cultured at 37°C under 5% CO_2_ atmosphere in DMEM and RPMI-1640 medium, respectively. Media purchased from Gibco (Life Technologies Co.) were supplemented with 10% fetal bovine serum (Sigma-Aldrich) and 1% penicillin/streptomycin (100 units/mL).

### Virus production

Virions used in the study consisted of HIV-1 core particles (NL4-3 strain) pseudotyped with the VSV-G envelope. Viral stocks were produced by calcium phosphate-mediated cotransfection of HEK293T cells with the pMD.G plasmid encoding a VSV-G envelope and the NLENG1-ES-IRES vector (wt) or a pLevD mutant, as previously described (49, 64). Virus-containing supernatant was collected 48 h after cotransfection. To avoid plasmid DNA contamination, 100 µL of supernatant was treated with 10 U of benzonase nuclease (Sigma-Aldrich) at 37°C for 30 min. The concentration of HIV-1 p24 antigen in each virus preparation was determined using a commercial enzyme-linked immunosorbent assay (Perkin Elmer). Aliquots of virus preparations were stored at –80°C.

### Quantification of viral RNA

RNA was isolated from 10 µL of virus preparation using the RNeasy Plus Mini kit (Qiagen) and the QIAshredder homogenizer (Qiagen), according to the manufacturer’s instructions. Quantification of packaged gRNA was performed by real-time one-step RT-PCR with a Light Cycler instrument (Roche Life Science) using the La 9 (5′-GACGCTCTCGCACCCATCTC-3′) and La 8.1 (5′-CTGAAGCGCGCACGGCAA-3′) primers associated with the La TM probe (5′-TAGCCTCCGCTAGTCAAAATTTTTGGCGTXT-3′, modified with 6-carboxyfluorescein at the 5′-end and phosphorylated at the 3′-end; X corresponds to 5-carboxytetramethylrhodamine group) as previously described (65). Each sample was analyzed in duplicate.

### Infectivity assay

To obtain a multiplicity of infection (m.o.i.) reaching 0.2, a benzonase nuclease-treated virus preparation corresponding to 20 ng of p24 antigen was inoculated to 5 × 10^5^ MT4 T cells. In the case of control assays, 1 h before infection, an inhibitor of reverse transcriptase (Efavirenz, EFV) or integrase (dolutegravir, DTG) was added to the culture medium at a final concentration of 1 µM. One, 2, and 3 days after infection, GFP expression, which was related to the percentage and mean fluorescence intensity (MFI) of green fluorescent protein-positive (GFP+) cells, was estimated by flow cytometry using a FACSCelesta flow cytometer (BD Biosciences).

### Quantification of reverse transcription products

We inoculated 5 × 10^5^ MT4 T cells with a benzonase nuclease-treated virus preparation corresponding to 20 ng of p24 antigen. DNA was isolated from infected cells using the QIAamp DNA blood mini kit (Qiagen), as described in several studies (35, 52), and quantified with a NanoVue spectrophotometer (GE Healthcare). We quantified the reverse transcription products by real-time PCR using a Light Cycler instrument (Roche Life Science). Copy numbers of DNA species were determined from calibration curves obtained by amplifying pre-determined amounts (from 25 to 25.10^6^ copies) of a plasmid containing the targeted sequences. The FwSS(-) (5′ATCTGAGCCTGGGAGCTCTCT-3′) and AA55 (5′CTGCTAGAGATTTTCCACACTGAC-3′) primers associated with the MLC1MGBLTR probe (FAAGT+AGTGTG + TGCCCQ, F and Q correspond to 6-carboxyfluorescein and TAMRA, respectively; +A and +T correspond to LNA nucleotides) were used to quantify the minus-strand strong-stop DNA as reported in previous studies (4, 66). The U3NL9496 (5′GCTGCATATAAGCAGCTGCTTTTTGCCT3′) and AA55 primers associated with the MLC1MGBLTR probe were used to quantify the minus-strand DNA after the first strand transfer as previously described (66). The MH531 (5′TGTGTGCCCGTCTGTTGTGT3′) and MH532 (5′GAGTCCTGCGTCGAGAGATC3′) primers associated with the MH FL (5′CCCTCAGACCCTTTTAGTCAGTGTGGAAFL3′, FL corresponds to fluorescein) and MH LC (5′LC-TCTCTAGCAGTGGCGCCCGAACAG3′Ph, LC, and Ph correspond to LC red 640 dye and phosphorylated, respectively) probes were used to quantify the late HIV-1 DNA products as previously reported (67, 68).

## Data Availability

The authors confirm that the data supporting the findings of this study are available within the article and its supplemental material.

## SUPPLEMENTAL MATERIAL

Supplemental figures jvi.01840-25-s0001.pdf Figures S1 to S5.

Table S1 jvi.01840-25-s0002.pdf Oligodeoxyribonucleotides used in site-directed mutagenesis by PCR.

ASM does not own the copyrights to Supplemental Material that may be linked to, or accessed through, an article. The authors have granted ASM a non-exclusive, world-wide license to publish the Supplemental Material files. Please contact the corresponding author directly for reuse.
