# Supplementary material for: Effect of TAR hairpin stabilization on HIV-1 reverse transcription
Source: J Virol. 2026 Apr 29;100(5):e01840-25. doi: 10.1128/jvi.01840-25 (PMC13185573; doi:10.1128/jvi.01840-25)
Supplement: Supplemental figures — Figures S1 to S5. [file jvi.01840-25-s0001.pdf]

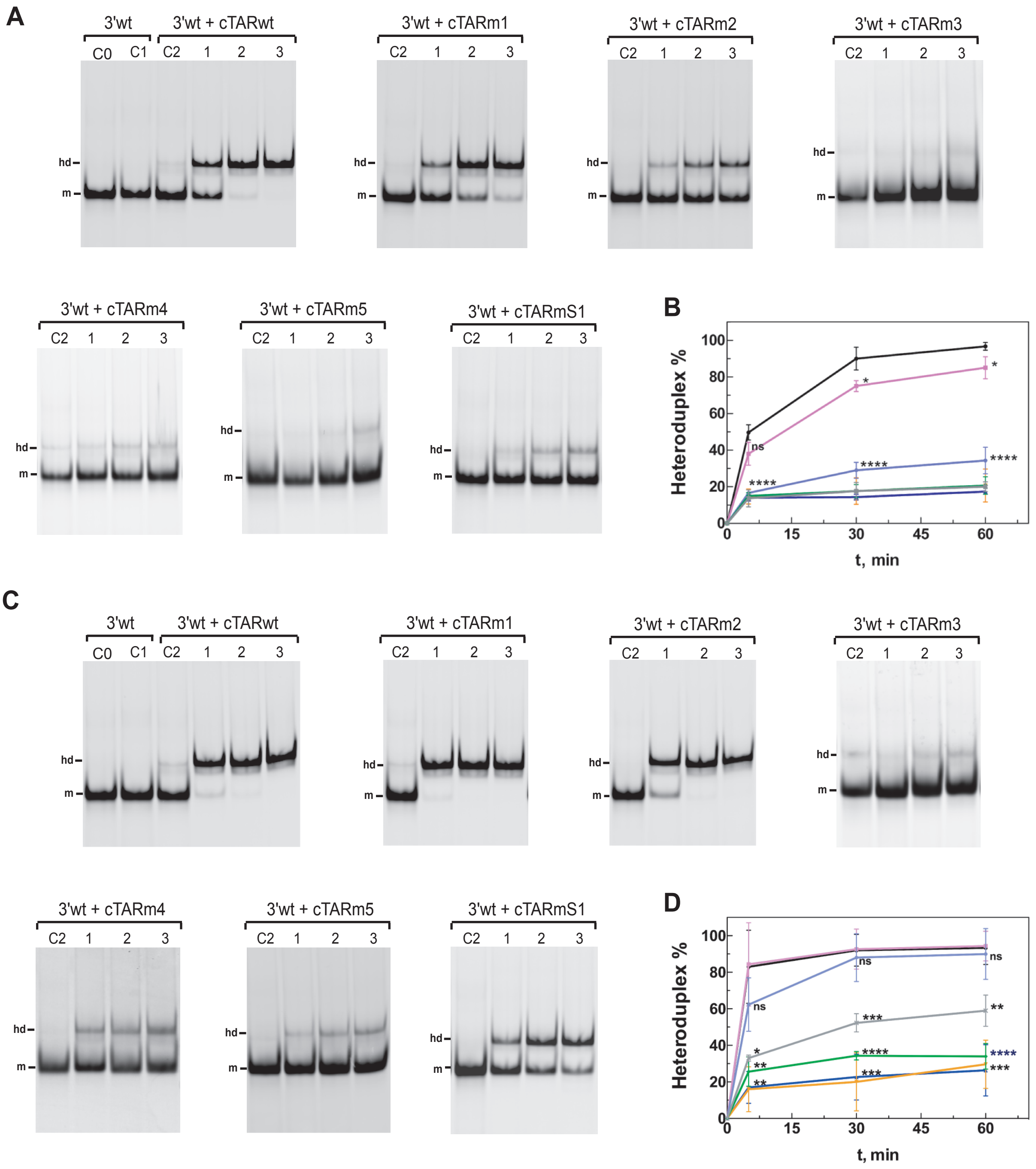

A
3'wt
3'wt + cTARwt
C0
C1
C2
1
2
3
hd
m
3'wt + cTARm1
3'wt + cTARm2
3'wt + cTARm3
3'wt + cTARm4
3'wt + cTARm5
3'wt + cTARmS1
B
Heteroduplex %
t, min
0
15
30
45
60
20
40
80
100
ns
*
****
C
D
***
**

FIG S1 Mutational analysis of TAR DNA–RNA annealing in the presence of NC. The NC-mediated annealing assays were performed as described in Materials and Methods. (**A**) Assays with NC were carried out at a protein to nucleotide molar ratio of 1:12. Lane C0, a heat-denatured control, was used to identify the band corresponding to the monomeric 3' TARwt RNA that was labeled at its 5'-end by the Cy®5.5 fluorescent dye. Lane C1, the 5'-end labeled 3' TARwt RNA was incubated for 60 min in the presence of NC. Lanes C2, the 5'-end labeled 3' TARwt RNA was incubated with the wild-type or mutant cTAR DNA for 60 min in the absence of NC. Lanes 1-3, the 5'-end labeled 3' TARwt RNA was incubated with the wild-type or mutant cTAR DNA for 5, 30 or 60 min in the presence of NC. The monomeric and heteroduplex forms of 3' TARwt are indicated by m and hd, respectively. (**B**) Percentage of the cTAR mutant–3' TARwt heteroduplex in the presence of NC at a protein to nucleotide molar ratio of 1:12. Black line, cTARwt; purple line, cTARm1; indigo line, cTARm2; blue line, cTARm3; green line, cTARm4; orange line, cTARm5; gray line, cTARmS1. Data represent the mean ± SD of at least three independent experiments. Means were compared with those of the wild type. *, $p<0.05$; **, $p<0.01$; ***, $p<0.005$; ****, $p<0.001$; ns, not statistically significant (unpaired two-samples t test). (**C**) Assays with NC were carried out at a protein to nucleotide molar ratio of 1:7. (**D**) Percentage of the cTAR mutant–3' TARwt heteroduplex in the presence of NC at a protein to nucleotide molar ratio of 1:7.

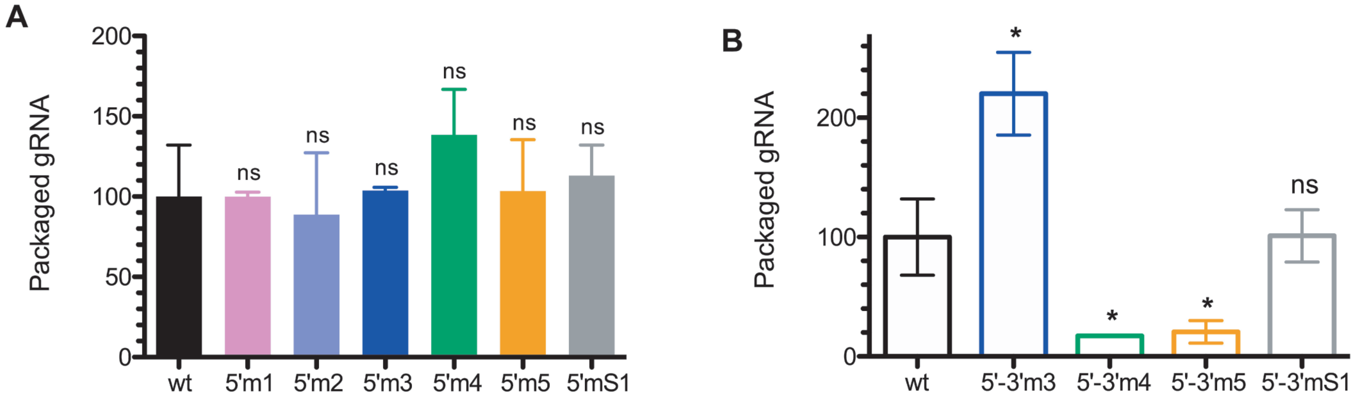
A
Packaged gRNA
200
150
100
50
0
ns
ns
ns
ns
ns
ns
wt
5'm1
5'm2
5'm3
5'm4
5'm5
5'mS1
B
Packaged gRNA
200
100
0
*
*
*
ns
wt
5'-3'm3
5'-3'm4
5'-3'm5
5'-3'mS1

FIG S2 Effect of single (**A**) and double (**B**) TAR mutations on RNA packaging. The level of the packaged gRNA (per CAp24 equivalents) was normalized to the wild-type (arbitrarily set to 100%). Data represent the mean ± SD of at least two independent experiments. Means were compared with those of the wild type. *, $p<0.05$; ns, not statistically significant (unpaired two-samples t test).

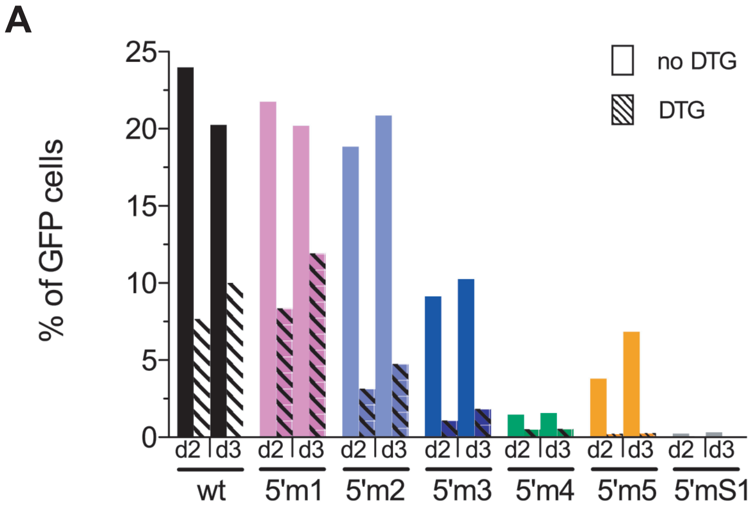

A
25
20
15
10
5
0
% of GFP cells
no DTG
DTG
d2 d3 d2 d3 d2 d3 d2 d3 d2 d3 d2 d3 d2 d3
wt
5'm1
5'm2
5'm3
5'm4
5'm5
5'mS1

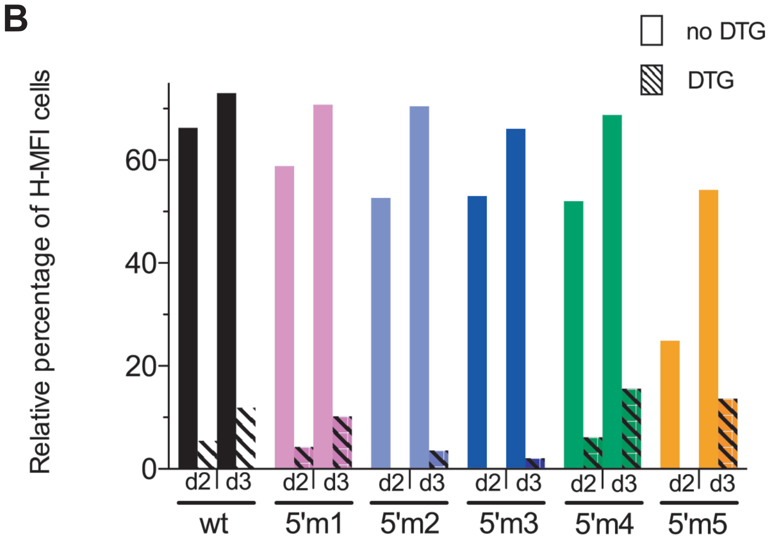

B
no DTG
DTG
60
40
20
0
Relative percentage of H-MFI cells
d2 d3 d2 d3 d2 d3 d2 d3 d2 d3 d2 d3
wt
5'm1
5'm2
5'm3
5'm4
5'm5

FIG S3 Effect of dolutegravir on time course of GFP expression. MT4 T-cells were infected with either HIV-1 *env*$^{-}$ *gfp*$^{+}$ harboring the wild-type TAR sequences or a mutant 5’ TAR sequence, each pseudotyped with VSV-G protein, in the absence (solid bars) or presence of dolutegravir (hatched bars). Two (d2) and three (d3) days postinfection, flow cytometry was used to determine the percentage of GFP+ cells and the mean of fluorescence intensity (MFI) of GFP expression. (**A**) Percentage of GFP+ cells (percentage of L-MFI cells + percentage of H-MFI cells). Data are representatives of independent experiments. (**B**) Relative percentages of H-MFI cells in the populations of GFP+ cells were calculated from data presented in (A). The relative percentages of H-MFI cells infected with the 5’ TARmS1 mutant are not indicated because the raw data in (A) are too low to be considered.

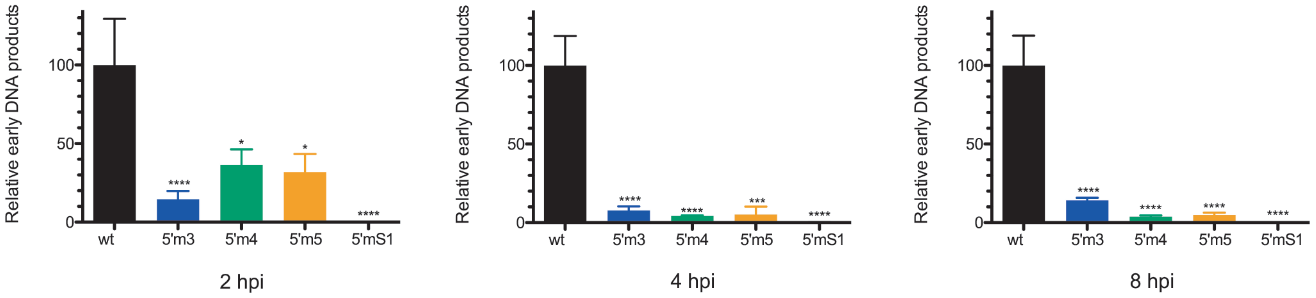
Relative early DNA products
100
50
0
wt
5'm3
5'm4
5'm5
5'mS1
****
*
*
****
2 hpi
Relative early DNA products
100
50
0
wt
5'm3
5'm4
5'm5
5'mS1
****
****
***
****
4 hpi
Relative early DNA products
100
50
0
wt
5'm3
5'm4
5'm5
5'mS1
****
****
****
****
8 hpi

FIG S4 Effect of single TAR mutations on strong-stop DNA synthesis. MT4 T-cells were infected with equivalent amounts of wild-type or mutant viruses as described in Materials and Methods. At 2, 4 or 8 h postinfection (2, 4 or 8 hpi), cells were harvested for DNA extraction and early (strong-stop) DNA was measured by qPCR. The levels of early DNA products were normalized to the wild-type (arbitrarily set to 100%). Data represent the mean ± SD of at least three independent experiments. Means were compared with those of the wild type. *, $p<0.05$; **, $p<0.01$; ***, $p<0.005$; ****, $p<0.001$; ns, not statistically significant (unpaired two-samples t test).

A

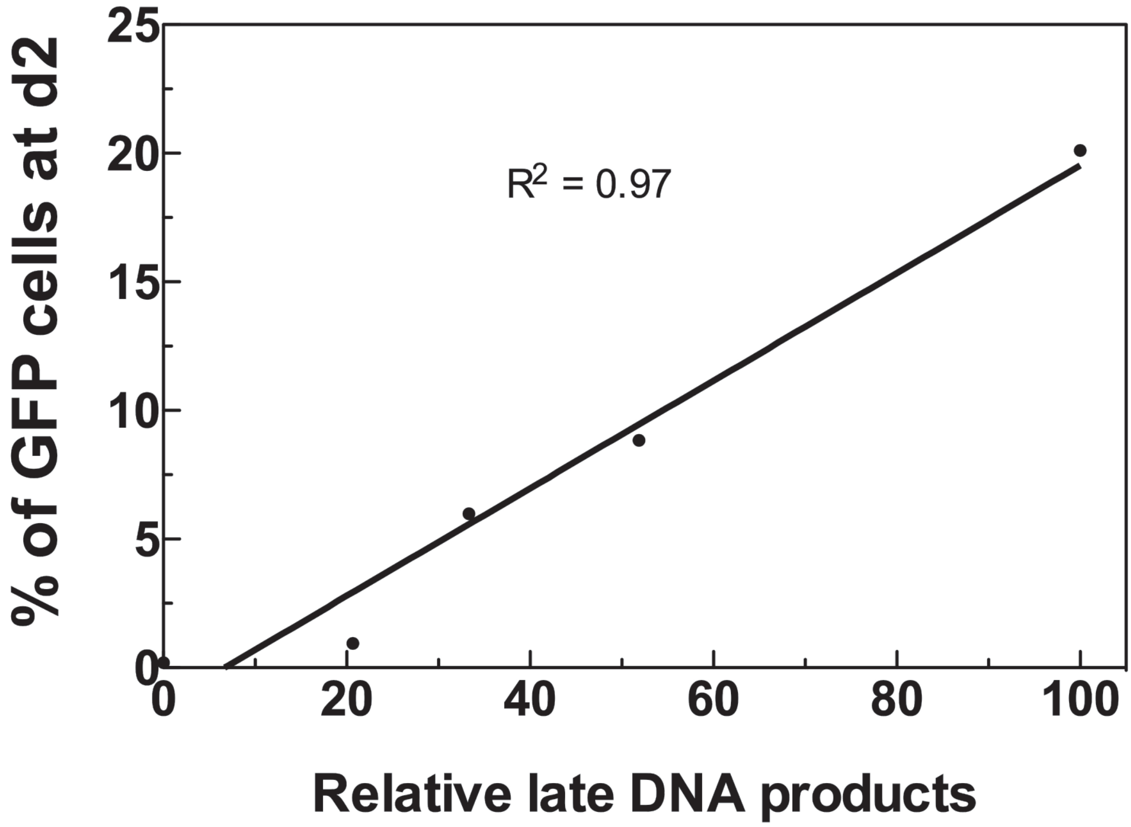

% of GFP cells at d2
25
20
15
10
5
0
0
20
40
60
80
100
Relative late DNA products
R2 = 0.97

B

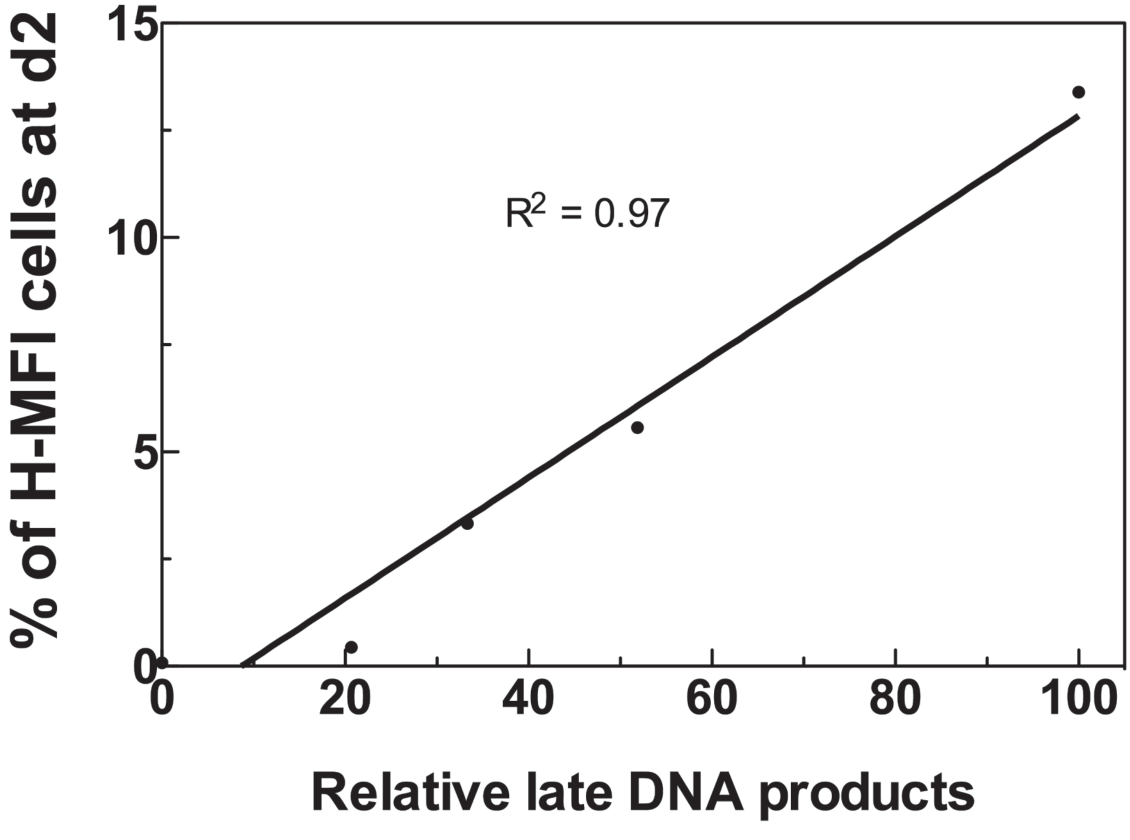

% of H-MFI cells at d2
15
10
5
0
0
20
40
60
80
100
Relative late DNA products
R2 = 0.97

FIG S5 The percentages of GFP cells (A) and H-MFI cells (B) at d2 are proportional to the amount of viral DNA. The linear regressions were drawn with the d2 data from Figure 7 and the qPCR data from Figure 8C (right panel, 24 hpi).
