# Supplementary material for: Effect of TAR hairpin stabilization on HIV-1 reverse transcription
Source: J Virol. 2026 Apr 29;100(5):e01840-25. doi: 10.1128/jvi.01840-25 (PMC13185573; doi:10.1128/jvi.01840-25)
Supplement: Table S1 — Oligodeoxyribonucleotides used in site-directed mutagenesis by PCR. [file jvi.01840-25-s0002.pdf]

Primer name	5'-3' primer sequence	Direction	Template	Purpose
OXho<br>OEco	GCACTCGAGGACGTCTAAGAAACCATTATTATC<br>GCAGAATTCGCGCGCTTCAGCAAG	Fwd<br>Rev	NLENG1-ES-IRES	pLF1
Owt(+)<br>Om1	CGGCACGAGCTCTCTGGCTAACTAGG<br>GACGAGCTCCCAGGCTCAGATCTGGCTAACCAGAGAGAC	Fwd<br>Rev	pLF1	pLF1-m1
Om2<br>Om1	GCAGAGCTCTCTGGCTAACCAGGGAACCCACTGC<br>GACGAGCTCCCAGGCTCAGATCTGGCTAACCAGAGAGAC	Fwd<br>Rev	pLF1	pLF1-m2
Om3<br>Owt(-)	GCAGAGCTCTCTGGCTAACTAGAGAGACCACTGCTTAAGCCTC<br>GCCTGGGAGCTCCCAGGCTCAG	Fwd<br>Rev	pLF1	pLF1-m3
Om4-fwd<br>Om4-rev	CGGCACGAGCTCTCTGGCTAACCAGGGAACCCACTG<br>GAGCTGAGCTCCCAGGCTCAGATCTGGTCTAACCAGGGAGACCCAGTACAG	Fwd<br>rev	pLF1	pLF1-m4
Om5-fwd<br>Om5-rev	CGTCACGAGCTCTCTGGTCTAACTAGGGAGACCCACTGCTTAAGCCTC<br>CCGCTCGTCGTGGGAGCTCCCAGGCTCAG	Fwd<br>Rev	pLF1	pLF1-m5
OmS-1fwd<br>OmS-1rev	CGGCACGAGCTCTCTGGCTAACTACTCTCACCCACTGCTTAAGCCTC<br>CATCGTGAGCTCCCAGGCTCAGATCTGGTCTAACCACCCTACCCAGTACAGGCAAAAAGC	Fwd<br>Rev	pLF1	pLF1-mS1
OXFW<br>OXREV	GGCTCGCTCGAGACCTAGAAAAACATG<br>CTATTGCTCGAGCAGCCTTTCCTGGTGTAAG	Fwd<br>Rev	NLENG1-ES-IRES	pEP2
Om3<br>Owt(-)	GCAGAGCTCTCTGGCTAACTAGAGAGACCACTGCTTAAGCCTC<br>GCCTGGGAGCTCCCAGGCTCAG	Fwd<br>Rev	pEP2	pEP2-m3
Om4-fwd<br>Om4-rev	CGGCACGAGCTCTCTGGCTAACCAGGGAACCCACTG<br>GAGCTGAGCTCCCAGGCTCAGATCTGGTCTAACCAGGGAGACCCAGTACAG	Fwd<br>Rev	pEP2	pEP2-m4
Om5-fwd<br>Om5-rev	CGTCACGAGCTCTCTGGTCTAACTAGGGAGACCCACTGCTTAAGCCTC<br>CCGCTCGTCGTGGGAGCTCCCAGGCTCAG	Fwd<br>Rev	pEP2	pEP2-m5
OmS-1fwd<br>OmS-1rev	CGGCACGAGCTCTCTGGCTAACTACTCTCACCCACTGCTTAAGCCTC<br>CATCGTGAGCTCCCAGGCTCAGATCTGGTCTAACCACCCTACCCAGTACAGGCAAAAAGC	Fwd<br>Rev	pEP2	pEP2-mS1

Fwd: forward primer – Rev: reverse primer

Table S1. Oligodeoxyribonucleotides used in site-directed mutagenesis by PCR
